# Determinants of COVID-19 knowledge and self-action among African women: Evidence from Burkina Faso, the Democratic Republic of Congo, Kenya, and Nigeria

**DOI:** 10.1371/journal.pgph.0001688

**Published:** 2023-05-03

**Authors:** Joseph A. Braimah, Vincent Z. Kuuire, Elijah Bisung, Mildred M. K. Pagra, Moses M. Kansanga, Bradley P. Stoner

**Affiliations:** 1 Department of Health & Society, University of Toronto Scarborough, Toronto, Canada; 2 Department of Geography, Geomatics and Environment, University of Toronto Mississauga, Mississauga, Canada; 3 Social and Behavioural Health Sciences Division, Dalla Lana School of Public Health, University of Toronto, Toronto, Canada; 4 School of Kinesiology and Health Studies, Queen’s University, Kingston, Canada; 5 Department of African and General Studies, SD Dombo University of Business & Integrated Development Studies, Wa, Ghana; 6 Department of Geography, George Washington University, Washington, DC, United States of America; 7 Department of Public Health Sciences, Queen’s University, Kingston, Canada; University of Michigan, UNITED STATES

## Abstract

Knowledge of infectious diseases and self-action are vital to disease control and prevention. Yet, little is known about the factors associated with knowledge of and self-action to prevent the coronavirus disease (COVID-19). This study accomplishes two objectives. Firstly, we examine the determinants of COVID-19 knowledge and preventive knowledge among women in four sub-Saharan African countries (Kenya, Nigeria, the Democratic Republic of Congo, and Burkina Faso). Secondly, we explore the factors associated with self-action to prevent COVID-19 infections among these women. Data for the study are from the Performance for Monitoring Action COVID-19 Survey, conducted in June and July 2020 among women aged 15–49. Data were analysed using linear regression technique. The study found high COVID-19 knowledge, preventive knowledge, and self-action among women in these four countries. Additionally, we found that age, marital status, education, location, level of COVID-19 information, knowledge of COVID-19 call centre, receipt of COVID-19 information from authorities, trust in authorities, and trust in social media influence COVID-19 knowledge, preventive knowledge, and self-action. We discuss the policy implications of our findings.

## Introduction

In the early stages of the coronavirus disease (COVID-19) pandemic, experts warned that low- and middle-income countries (LMICs), including those in sub-Saharan Africa (SSA), could become the epicentres of the pandemic [1, 2]. There were suggestions that physical distancing, an effective strategy for reducing the spread of the virus, may not work effectively in SSA because of crowding, inadequate housing, poor city planning, and other poor living conditions that characterize the living arrangements of millions of people in the subregion [1, 3]. Yet, relative to other locations in the world, SSA has been spared the worse ravages of the COVID-19 pandemic so far [4]. Nonetheless, the prevailing realities of inadequate and dysfunctional health systems together with the low vaccine availability and uptake in the subregion create a situation where even marginal increases in COVID-19 cases could overwhelm health systems—a situation experts fear could still be a possibility in the event of new and more infectious strains of the virus [2, 5]. Some of these concerns are bolstered by the rapid evolution of the disease’s epidemiology and current changes to its responses worldwide. In the wake of these fears, public health officials are reemphasizing messages that aim to deliver valuable and accurate information about COVID-19 to ensure self-action that contributes to fewer infections.

Empirical epidemiological findings suggest that accurate disease preventive knowledge and self-action are vital to controlling and preventing their spread [6, 7]. For example, accurate knowledge aids in eradicating myths and misconceptions about diseases (e.g., Ebola virus disease and COVID-19) [8, 9]. It is also helpful in shaping attitudes and promoting appropriate preventive practices such as physical distancing, regular handwashing, and vaccination [10]. In the context of COVID-19, accurate knowledge is essential for identifying COVID-19 symptoms and distinguishing them from other diseases.

Following the declaration of COVID-19 as a global pandemic, studies have explored knowledge, attitudes, and practices toward the virus [9, 11]. So far, the burgeoning literature from SSA reports low knowledge, misconception, and misinformation about COVID-19, which collectively result in low self-action [12, 13]. For instance, COVID-19 was considered a “white man’s disease” by a section of Ugandan men during its earlier stages, a sentiment that adversely impacted response against it [13]. Notwithstanding the usefulness of these studies in enhancing our understanding of COVID-19 and its containment, they are limited in three major ways. Firstly, they focus mainly on knowledge and attitudes, which are necessary but insufficient to curb the pandemic. Secondly, whereas earlier studies allude to the significance of location in shaping health and health behaviours [14], existing studies on COVID-19 in the subregion fail to account for the influence of place of residence on women’s knowledge, preventive knowledge, and action toward it. Lastly, existing studies focus on the general population and, hence, are limited in highlighting determinants of women’s COVID-19 knowledge and self-action. Addressing these research gaps will imperatively provide empirical evidence for contextual public health programs necessary to contain and prevent COVID-19.

In this study we examine factors associated with COVID-19 knowledge and preventive knowledge among women in four countries in SSA (Burkina Faso, the Democratic Republic of Congo (DRC), Kenya, and Nigeria). We also draw on the Health Belief Model (HBM) to explain factors that influence these women’s actions aimed at preventing the disease. In the context of SSA, accurate COVID-19 preventative knowledge and proper self-action are critical, especially given that an overwhelming proportion of populations exist on the disadvantaged side of the global vaccine accessibility and the widespread vaccine apathy [15, 16].

The HBM, developed in the 1950s to explain and predict health behaviours and outcomes, posits that a person’s behaviour or action toward a health problem is influenced by the perceived threat of the disease or illness and the effectiveness of recommended health action(s) [17]. The HBM consists of six interacting constructs, namely, perceived susceptibility, perceived severity, perceived benefits of action, existing barriers, cues to act, and modifying factors [18, 19]. In the context of this study, we contend that women’s self-action to prevent COVID-19 infection will depend on their perceived susceptibility to the virus, its perceived severity, how beneficial they think recommended COVID-19 public health measures are, their appraisal of existing obstacles to act, their beliefs about the benefits associated with acting, and their self-efficacy. For instance, women’s perceived susceptibility or subjective view of the risk of being infected by COVID-19 and associated benefits can influence their knowledge and propensity to act, and this may be a function of their age, access to COVID-19 information and place of residence [7]. Their level of education and level of trust in COVID-19 information also have the potential to shape their perception about the benefits of taking action [6]. Furthermore, where they reside and where they receive COVID-19 information from have the potential to shape their knowledge and decisions to adhere to COVID-19 public health messages and containment measures [20, 21]. Likewise, women’s socioeconomic characteristics and place of residence have potential impacts on their self-efficacy and subsequently their behaviour [20, 21].

The HBM is one of the earliest and most widely used psychosocial models for explaining and predicting health-related knowledge and behaviours [21, 22]. In sub-Saharan Africa, the model has been used to understand and predict health behaviours towards various infectious diseases, including Ebola virus disease [7], COVID-19 [23], and HIV/AIDS [21]. The use of the HBM in this study helps to explain factors that influence women’s preventive actions toward COVID-19. In an era of evidence-based practice, findings from this study will not only augment existing literature on COVID-19 but will contribute to prevention programs, especially with the evolving nature of the COVID-19 variants.

### Determinants of knowledge of infectious diseases

Empirical research highlight a range of socioeconomic and demographic factors that influence people’s knowledge of infectious diseases [11, 24, 25]. For instance, knowledge of infectious diseases has been found to differ by age [24, 25], partly because younger adults often have easy and better access to information on infectious diseases than older people. Knowledge of infectious diseases is also linked to level of education [6, 11]. This is because education enhances access to and comprehension of accurate information about diseases [11]. In the case of COVID-19, social and print media have emerged as invaluable sources of information, and these are more easily accessible to educated individuals [26]. Income also has an effect on knowledge of infectious diseases, given its ability to empower people to seek information [24, 27]. In addition to socioeconomic and demographic factors, a substantial amount of literature links knowledge of infectious diseases to place of residence [11, 28]. Specifically, studies have consistently reported higher knowledge scores on infectious diseases among urban dwellers than their rural counterparts [11, 28]. Several reasons account for this disparity, including the presence of more accessible sources of information in urban areas and the relatively higher educational levels of urban residents [28]. People’s trust in and the source of information on infectious diseases can also influence their knowledge levels [29, 30].

### Determinants of preventive knowledge of infectious diseases

Pervasive misinformation and misconceptions about diseases render accurate preventive knowledge crucial to curbing their spread [8, 26]. Albeit limited, the literature reveals several factors that determine accurate preventive knowledge of infectious diseases. Firstly, in Saudi Arabia, Baig and colleagues found that older people were more likely to report misconceptions about COVID-19 and its prevention than younger people [6]. A similar observation was made among ever-married women in Bangladesh regarding HIV infections [31]. Older people tend to have less access to health information than younger adults. Education is also associated with people’s knowledge on preventing infectious diseases [6, 24, 28]. Education facilitates access and evaluation of accurate information on infectious diseases prevention [30]. It is thus useful in expelling misconceptions about preventing infectious diseases. Other identified factors that influence preventive knowledge of infectious diseases include income [32, 33], place of residence [24, 28], trust [34, 35], and source of information [30, 35].

### Determinants of self-action on infectious diseases

The literature identifies various interrelated factors that shape people’s behaviours toward infectious diseases, which are thus important to consider in the context of COVID-19 in SSA. For example, age is a major determining factor in preventive action against infectious diseases [24, 36]. This is because aging associated functional limitation can hinder older adults’ ability to act to prevent infections [24, 36]. Conversely, some studies suggest that younger adults are less likely to act because of their perceived immunity and high-risk-taking attitudes [25, 37]. Education also dictates actions toward infectious diseases as it enhances access to accurate information and knowledge on diseases, including the need to act [7, 38]. Furthermore, income has the potential to empower people to engage in preventive actions [24]. For instance, regular handwashing with soap and face masking among others, which are important COVID-19 preventive measures can be challenging to low-income earners. Paradoxically, a study on knowledge, attitudes, and practices about influenza in South Africa found individuals with higher household incomes to be less likely to get vaccinated [39]. Place of residence also has implications for action against infectious diseases. For example, urban dwellers have been found to have higher practice scores on COVID-19 than their rural counterparts [10, 40]. This is because urban residents have access to more infectious disease prevention information than their rural counterparts. Also, the better infrastructure in urban areas can facilitate infectious disease prevention practices such as regular hand washing. Trust in information and the source of information also determine the actions people take [29, 35, 39]. It is argued that risk communication about diseases by trusted persons in an honest, consistent, and coordinated manner motivates people to take act [34, 35].

Using Burkina Faso, DRC, Kenya, and Nigeria as case studies, we examine the determinants of COVID-19 knowledge, preventive knowledge, and self-action among women in SSA. These countries, notwithstanding their relatively low COVID-19 rates, serve as useful case studies because they exhibit stark in-country geographic, economic, and health disparities [41, 42]. For example, the multidimensional poverty index (MPI) ―referred to as the percentage of the population that is multidimensionally poor adjusted by the intensity of the deprivations ―in Burkina Faso in 2020 ranged from 0.214 in urban areas to 0.604 in rural areas [43]. The highest regional MPI incidence in Burkina Faso is in the Est region (0.660), while the lowest is in the Centre region (0.197). Nigeria has a national MPI of 0.254, with the highest in Kebbi state (0.585) and the lowest in Lagos state (0.016) [44]. Kenya’s MPI ranges from 0.084 in urban areas to 0.226 in rural areas, while that of the DRC range from 0.166 in urban areas to 0.460 in rural areas. As of January 2023, the Africa CDC COVID-19 Dashboard reported the total number of deaths from the disease to be 5,688 in Kenya, 3,155 in Nigeria, 387 in Burkina Faso, and 1,460 in the DRC [45]. With the potential rise in COVID-19 cases and the emergence of new variants, empirical evidence that will inform public health practices is critical.

## Materials and methods

### Data

The data for this study are from the Performance Monitoring for Action (PMA) COVID-19 survey conducted in Kenya [46], Burkina Faso [47], the DRC (Kinshasa) [48], and Nigeria (Lagos and Kano) [49] in June and July 2020 among females aged 15–49 years. The PMA project collects longitudinal and cross-sectional data from women on a variety of family planning and health issues, including abortion, maternal and child health, primary health care and COVID-19, in 9 countries in Africa and Asia. The goal of PMA is to provide actionable data to guide policy and programming. Data collection is done by trained female data collectors under the auspices of selected local universities and research organizations. Funding for PMA is from the Bill & Melinda Gates Foundation. The PMA employs a two-stage cluster design in sampling respondents. Samples are typically stratified by rural-urban residence and region of residence. The PMA used geographical clusters called Enumeration Areas (EAs), provided by the statistical agency in each country as its sampling frame. A random sample of households is then drawn from each enumeration area for interviewing. All eligible females in sampled households are interviewed with their consent. The COVID-19 survey was phone-based and among women who had participated in a baseline survey in November and December 2019 as part of a longitudinal panel data. A total of 3,415, 5,952, 5,952, and 1,299 women from Burkina Faso, Kenya, the DRC, and Nigeria, respectively, make up our analytical sample.

### Measures

#### Dependent variables

The dependent variables for the study are COVID-19 knowledge, preventive knowledge, and self-action. These three variables are continuous and are derived from a set of questions related to knowledge and self-action toward Covid-19. *COVID-19 knowledge* was derived from nine relevant questions about COVID-19 infection, and the questions had “yes/no” responses. Respondents were asked if certain statements about COVID-19 infections are true. Some of the statements are that; 1) some people cannot be infected with Coronavirus; 2) most people experience mild or no symptoms; and 3) only people with symptoms are contagious. A value of “1” is assigned if a correct response is provided and “0” if a wrong answer is given. Similarly, the variable *preventive knowledge* was derived from yes/no responses to 10 questions on various actions that respondents think can reduce the risk of being infected (1 = if answered correctly; 0 = if answered incorrectly). These actions include washing hands with soap and water frequently, washing hands with hand sanitizer frequently, avoiding close contacts (2 meters) with people when one gets out, prayer, and avoiding shaking hands with others. The variable *self-action* was also generated from 10 questions on the actions respondents took to prevent becoming infected, coded as yes (1 = yes) if respondents took the particular action and no (0 = no) if respondents did not take the particular action. These actions include washing hands with soap and water frequently, washing hands with hand sanitizer, staying in your home, getting vaccinated, and prayer. We computed a summative variable for each of the three sets of variables following an internal consistency reliability analysis which produced Cronbach’s alpha of 0.7, 0.9, 0.8 for COVID-19 knowledge, preventive knowledge, and self-action, respectively.

#### Independent variables

Four groups of variables were introduced in our analysis as covariates, namely socio-demographic, locational, COVID-19 knowledge source, and trust. These variables were informed by the literature and the HBM [11, 17, 27, 28]. Our socio-demographic variables include age, marital status, and level of education. Locational variables include rural-urban residence and region of residence. COVID-19 information source variables include Knowing or hearing of call center. The others are hearing about COVID-19 from authorities, family and friends, traditional media, and social media. All COVID-19 knowledge source variables are dummy variables. Our final group of explanatory variables are on trust. Respondents were asked if they trust COVID-19 information from the different sources, including family and friends, government authorities, traditional media, and social media.

### Analytical techniques

Two separate analyses were conducted in this study. We first performed univariate analysis to describe the sample distribution. Second, we employed linear regression to examine how socio-demographic, source of information, trust, and place-based factors influence our outcomes of interest. We used linear regressions due to the continuous nature of our independent variables. We accounted for sociodemographic factors in model 1. In model 2, we introduced locational factors. Model 3 accounted for the variables on COVID-19 information sources, while model 4 accounted for trust factors. Regression coefficients are standardized for easy and more informative interpretations. Sampling weights provided in the data were applied to all the analyses.

### Ethical considerations

Data for this study is secondary and were collected and made publicly available by the PMA project. The PMA project received approval for the study from relevant ethics review boards in the respective countries and the Johns Hopkins Bloomberg School of Public Health Institutional Review Board. All participants consented to the study.

## Results

### Descriptive results

Table 1 shows the characteristics of our analytical samples. The mean COVID-19 knowledge level ranged from 5.1 (SD = 1.47) in the DRC to 6.7 (SD = 1.25) in Kenya. The mean COVID-19 prevention knowledge ranged from 5.6 (SD = 1.77) in the DRC to 6 (SD = 1.6) in Nigeria. For COVID-19 self-action, the highest mean score is recorded in Kenya (6.3, SD = 1.02), with the lowest in the DRC (5.6, SD = 1.55). Except for Nigeria (33.7%), most study respondents across the study countries (Kenya = 38.7%; Burkina Faso = 39.4%; the DRC = 41.5%) were aged from 21 to 30 years. In terms of rural-urban residence, most respondents in Burkina Faso (75.5%) and Nigeria (89.6%) were from urban areas while most respondents in Kenya lived in rural areas (61.8%). Furthermore, an overwhelming majority of respondents reported having a lot of COVID-19 knowledge, with the DRC (95%) reporting the highest proportion, followed by Burkina Faso (93.2%), Kenya (86%), and Nigeria (79.6%). Relatedly, few respondents are willing to disclose a family member’s COVID-19 status. Specifically, 14.9%, 4.8%, 11.6%, and 12.5% of respondents in the DRC, Nigeria, Burkina, and Kenya, respectively, indicated that they would keep it a secret if a member of their family contracts COVID-19. Except for Nigeria (27.5%), nearly half of respondents from Burkina Faso (50.1%), Kenya (51.3%), and the DRC (44.6%) knew or heard of a COVID-19 call centre and the phone number to the call centre.

**Table 1:** Sample characteristics.

	Burkina Faso (n = 3,415)	DRC (n = 5,952)	Kenya (n = 5,952)		Nigeria (n = 1,299)	
Variables	Frequency (%)	Frequency (%)	Frequency (%)		Frequency (%)	
Covid-19 Knowledge*	6.0 (1.28) Min = 1; Max = 9	5.1 (1.47) Min = 0; Max = 9	6.7 (1.25) Min = 2; Max = 9		5.9 (1.5) Min = 0; Max = 9	
Preventive knowledge*	5.8 (1.4) Min = 0; Max = 8	5.6 (1.77) Min = 0; Max = 8	5.7 (1.4) Min = 0; Max = 8		6 (1.6) Min = 0; Max = 8	
Preventive action*	5.9 (SD = 1.14) Min = 1; Max = 8	5.6 (SD = 1.55) Min = 1; Max = 9	6.3 (SD = 1.02) Min = 2; Max = 8		6 (SD = 1.3) Min = 1; Max = 8	
**Age**						
15–20 years	597 (17.5)	232 (18.1)	696 (11.7)		192 (14.8)	
21–30 years	1,346 (39.4)	534 (41.5)	2,303 (38.7)		438 (33.7)	
31–40 years	993 (29.1)	318 (24.7)	1,880 (31.6)		441 (33.9	
41–50 years	479 (14)	202 (15.7)	1,073 (18)		228 (17.6)	
**Level of education**						
No formal education	1,144 (33.5)	5 (0.4)	119 (2)		71 (5.5)	
Primary/middle school	677 (19.8)	40 (3.1)	2,588 (43.5)		122 (9.4)	
Secondary/post primary	1,355 (39.7)	851 (66.2)	2,198 (36.9)		622 (47.9)	
Tertiary/post-secondary	239 (7)	390 (30.3)	1,047 (17.6)		484 (37.2)	
**Marital status**						
Never married	853 (24.5)	587 (45.6)	1,193 (20)		337 (25.9)	
Married/Co-habiting	2,368 (69.3)	604 (47)	4,068 (68.4)		848 (65.3)	
Divorced/Separated/Widowed	194 (5.7)	95 (7.4)	691 (11.6)		114 (8.8)	
**Rural/urban residence**						
Rural	837 (24.5)		3,676 (61.8)		135 (10.4)	
Urban	2,578 (75.5)		2,276 (38.2)		1,164 (89.6)	
**Region**			**County**		**State**	
Boucle du mouhoun	161 (4.7)		Bungoma	539 (9.1)	Lagos	946 (72.8)
Cascades	89 (2.6)		Kericho	609 (10.2)	Kano	353 (27.2)
Centre	1,182 (34.6)		Kiambu	525 (8.8)		
Centre-est	184 (5.4)		Kilifi	445 (7.5)		
Centre-nord	138 (4.0)		Kitui	646 (10.9)		
Centre-ouest	194 (5.7)		Nairobi	569 (9.6)		
Centre-sud	75 (2.2)		Nandi	864 (14.5)		
Est	808 (23.7)		Nyamira	466 (7.8)		
Hauts-bassins	86 (2.5)		Siaya	552 (9.3)		
Nord	291 (8.5)		Kakamega	481 (8.1)		
Plateau-central	132 (3.9)		West Pokot	256 (4.3)		
Sahel	41 (1.2)					
Sud-ouest	34 (1)					
**Covid-19 information**						
A little	14 (0.4)	10 (0.8)	215 (3.6)		88 (6.8)	
Some	220 (6.4)	55 (4.2)	619 (10.4)		177 (13.6)	
A lot	3,181 (93.2)	1,221 (95)	5,118 (86)		1,034 (79.6)	
**Keep family covid-19 secret**						
No	3,018 (88.4)	1,094 (85.1)	5,208 (87.5)		1,236 (95.2)	
Yes	397 (11.6)	192 (14.9)	744 (12.5)		63 (4.8)	
**Know or heard of call center**						
No	613 (18)	317 (24.6)	701 (11.8)		192 (14.8)	
Yes, knows the number	1,712 (50.1)	573 (44.6)	3,055 (51.3)		357 (27.5)	
Yes, but does not know the number	1,089 (31.9)	396 (30.8)	2,196 (37)		750 (57.7)	
**Authorities**						
No	1,796 (52.6)	785 (61)	3,589 (60.3)		984 (75.8)	
Yes	1,619 (47.4)	501 (39)	2,363 (39.7)		315 (24.2)	
**Family and friends**						
No	2,090 (61.2)	605(47.1)	3,035 (51)		560 (43.1)	
Yes	1,325 (38.8)	681 (52.9)	2,917 (49)		739 (56.9)	
**Traditional media**						
No	268 (7.9)	132 (10.3)	130 (2.2)		85 (6.5)	
Yes	3,147 (92.1)	1,154 (89.7)	5,822 (97.8)		1,214 (93.5)	
**Social media**						
No	2,841 (83.2)	917 (71.3)	3,026 (50.8)		579 (44.6)	
Yes	574 (16.8)	369 (28.7)	2,926 (49.2)		720 (55.4)	
**Trust in family and friends**						
No	938 (27.5)	825 (64.1)	2,981 (50.1)		863 (66.4)	
Yes	2,477 (72.5)	461 (35.9)	2,971 (49.9)		436 (33.6)	
**Trust in authorities**						
No	197 (5.8)	577 (44.9)	1,516 (25.5)		712 (54.8)	
Yes	3,218 (94.2)	709 (55.1)	4,436 (74.5)		587 (45.2)	
**Trust in traditional media**						
No	136 (4)	204 (15.9)	136 (2.3)		154 (11.9)	
Yes	3,279 (96)	1,082 (84.1)	5,816 (97.7)		1,145 (88.1)	
**Trust in social media**						
No	1,289 (37.8)	688 (53.5)	2,201 (37)		678 (52.2)	
Yes	2,126 (62.2)	598 (46.5)	3751 (63)		621 (47.8)	

#### Factors associated with COVID-19 knowledge

Tables 2–5 show findings from our multivariate regression analysis on factors associated with COVID-19 knowledge in Burkina Faso, the DRC, Kenya, and Nigeria, respectively. Detail results on factors associated with COVID-19 knowledge in Burkina Faso, the DRC, Kenya, and Nigeria can be found in S1–S4 Tables, respectively. The age of respondents was significantly associated with COVID-19 knowledge in Burkina Faso and Kenya. Specifically, women aged 31–40 years (β = 0.332, SE = 2.05, p < 0.05) and 41–50 years (β = 0.201, SE = 2.10, p < 0.05) in Burkina Faso and Kenya, respectively had significantly higher COVID-19 knowledge scores than those aged 15–20 years. Except for Nigeria, women’s level of education was associated with their COVID-19 knowledge scores. We observed significantly higher COVID-19 knowledge scores among women with tertiary/post-secondary level education in Burkina Faso (β = 1.150, SE = 6.12, p < 0.001), Kenya (β = 0.667, SE = 4.04, p < 0.001), and the DRC (β = 0.791, SE = 4.83, p < 0.001) than their counterparts with no formal education. On the other hand, in Nigeria, divorced, separated, or widowed (β = -0.533, SE = -2.61, p < 0.01) women had lower mean COVID-19 knowledge scores. Residing in urban areas was also associated with higher COVID-19 knowledge scores in Burkina Faso (β = 0.180, SE = 2.71, p < 0.01).

**Table 2:** Determinants of Covid-19 knowledge, preventive knowledge, and self-action in Burkina Faso.

	Covid-19 Knowledge	Preventive Knowledge	Covid-19 Self-action
	β (SE)	β (SE)	β (SE)
**Age**			
15–20 years (Ref)			
21–30 years	0.166 (1.14)	0.252 (1.44)	-0.169 (-1.66)
31–40 years	0.332 (2.05)*	0.492 (2.58)*	-0.093 (-0.84)
41–50 years	0.093 (0.53)	0.337 (1.48)	-0.213 (-1.81)
**Level of education**			
No formal education (Ref)			
Primary/middle school	0.058 (0.48)	-0.195 (-1.22)	-0.011 (-0.13)
Secondary/post primary	0.233 (1.41)	-0.352 (-2.02)*	-0.227 (-2.06)*
Tertiary/post-secondary	1.150 (6.12)***	-0.823 (-4.41)***	0.043 (0.34)
**Marital status**			
Never married (Ref)			
Married/Co-habiting	0.120 (0.61)	-0.298 (-1.52)	-0.150 (-1.31)
Divorced/Separated/Widowed	0.281 (1.12)	0.008 (0.03)	-0.245 (-1.29)
**Rural/urban residence**			
Rural (Ref)			
Urban	0.180 (2.71)**	-0.411 (-4.32)***	-0.066 (-1.07)
**County**			
Boucle du mouhoun (Ref)			
Cascades	-0.040 (-0.18)	-0.205 (-0.62)	-0.001 (-0.01)
Centre	-0.065 (-0.36)	-0.329 (-1.26)	0.579 (3.27)**
Centre-est	-0.623 (-1.91)	-1.102 (-3.71)***	0.555 (2.76)**
Centre-nord	0.398 (1.88)	0.110 (0.33)	0.524 (2.48)*
Centre-ouest	-0.393 (-1.79)	-0.934 (-2.78)**	0.132 (0.58)
Centre-sud	-0.148 (-0.60)	-0.794 (-2.13)*	0.385 (1.62)
Est	-0.024 (-0.13)	-0.446 (-1.55)	0.062 (0.34)
Hauts-bassins	0.043 (0.17)	-0.093 (-0.24)	-0.404 (-1.54)
Nord	-0.124 (-0.65)	-0.413 (-1.36)	0.458 (2.37)*
Plateau-central	0.006 (0.02)	0.038 (0.12)	0.479 (2.25)*
Sahel	-0.119 (-0.27)	0.343 (0.85)	-0.075 (-0.27)
Sud-ouest	-1.104 (-4.27)***	0.923 (2.09)*	-1.556 (-4.05)***
**Covid-19 information**			
A little (Ref)			
Some	0.204 (0.49)	-0.145 (-0.30)	0.200 (0.60)
A lot	0.174 (0.44)	-0.385 (-0.87)	0.161 (0.51)
**Keep covid-19 secret**			
No (Ref)			
Yes	-0.184 (-1.02)	0.356 (2.09)*	0.042 (0.36)
**Know or heard of call center**			
No (Ref)			
Yes, knows the number	0.356 (2.66)**	0.165 (0.90)	0.512 (5.20)***
Yes, but does not know the number	0.092 (0.79)	0.204 (1.43)	0.243 (2.87)**
**Authorities**			
No (Ref)			
Yes	0.015 (0.16)	-0.084 (-0.71)	0.093 (1.33)
**Family and friends**			
No (Ref)			
Yes	0.145 (1.71)	0.118 (1.00)	0.157 (0.157)*
**Traditional media**			
No (Ref)			
Yes	0.157 (1.17)	0.034 (0.19)	0.089 (0.79)
**Social media**			
No (Ref)			
Yes	-0.033 (-0.22)	-0.193 (-1.38)	0.047 (0.45)
**Trust in family and friends**			
No (Ref)			
Yes	-0.126 (-1.25)	0.124 (0.80)	0.024 (0.29)
**Trust in authorities**			
No (Ref)			
Yes	0.419 (1.47)	0.512 (2.30)*	0.187 (1.10)
**Trust in traditional media**			
No (Ref)			
Yes	0.361 (1.46)	-0.161 (-0.60)	0.089 (0.56)
**Trust in social media**			
No (Ref)			
Yes	0.099 (0.96)	0.460 (3.63)***	0.288 (3.87)***
Constant	4.245 (7.33)***	6.036 (9.71)***	4.768 (11.29)***
Observations	3415	3415	3415

β represents standardized coefficient

SE represents standard error

Constant ― also known as y-intercept is the mean of the dependent variable when all independent variables in the model are set to zero

* p < 0.05,

** p < 0.01,

*** p < 0.001

**Table 3:** Determinants of Covid-19 knowledge, preventive knowledge, and self-action in DRC.

	Covid-19 Knowledge	Preventive Knowledge	Covid-19 Self-action
	β (SE)	β (SE)	β (SE)
**Age**			
15–20 years (Ref)			
21–30 years	0.095 (1.11)	-0.066 (-0.71)	0.021 (0.33)
31–40 years	0.177 (1.92)	-0.118 (-1.19)	-0.044 (-0.62)
41–50 years	0.189 (1.92)	-0.064 (-0.61)	0.010 (0.14)
**Level of education**			
No formal education (Ref)			
Primary/middle school	0.269 (1.74)	-0.026 (-0.15)	0.313 (2.40)*
Secondary/post primary	0.416 (2.60)**	-0.065 (-0.36)	0.479 (3.54)***
Tertiary/post-secondary	0.791 (4.83)***	-0.204 (-1.12)	0.475 (3.47)***
**Marital status**			
Never married (Ref)			
Married/Co-habiting	0.015 (0.22)	-0.099 (-1.29)	0.017 (0.30)
Divorced/Separated/Widowed	-0.038 (-0.38)	-0.189 (-1.93)	-0.060 (-0.85)
**Covid-19 information**			
A little (Ref)			
Some	-0.149 (-0.98)	-0.152 (-0.92)	-0.102 (-0.90)
A lot	-0.062 (-0.46)	-0.018 (-0.12)	-0.052 (-0.49)
**Keep covid-19 secret**			
No (Ref)			
Yes	-0.117 (-1.61)	-0.065 (-0.79)	-0.210 (-2.94)**
**Know or heard of call center**			
No (Ref)			
Yes, knows the number	0.359 (4.54)***	0.013 (0.14)	0.286 (3.66)***
Yes, but does not know the number	0.322 (4.04)***	-0.064 (-0.69)	0.148 (1.95)
**Authorities**			
No (Ref)			
Yes	0.109 (2.11)*	-0.163 (-2.99)**	0.106 (2.77)**
**Family and friends**			
No (Ref)			
Yes	0.067 (1.40)	0.010 (0.17)	-0.024 (-0.63)
**Traditional media**			
No (Ref)			
Yes	0.351 (2.34)*	-0.258 (-1.19)	-0.014 (-0.12)
**Social media**			
No (Ref)			
Yes	0.111 (2.29)*	-0.206 (-3.72)***	0.137 (3.53)***
**Trust in family and friends**			
No (Ref)			
Yes	-0.094 (-1.64)	0.050 (0.82)	-0.097 (-2.50)*
**Trust in authorities**			
No (Ref)			
Yes	0.191 (2.87)**	0.316 (5.00)***	0.272 (6.05)***
**Trust in traditional media**			
No (Ref)			
Yes	-0.548 (-3.57)***	-0.141 (-0.74)	0.004 (0.03)
**Trust in social media**			
No (Ref)			
Yes	-0.050 (-0.88)	0.040 (0.63)	0.136 (3.28)**
Constant	5.917 (20.47)***	6.272 (19.91)***	5.434 (23.77)***
Observations	5952	5952	5952

β represents standardized coefficient

SE represents standard error

Constant ― also known as y-intercept is the mean of the dependent variable when all independent variables in the model are set to zero

* p < 0.05,

** p < 0.01,

*** p < 0.001

**Table 4:** Determinants of Covid-19 knowledge, preventive knowledge, and self-action in Kenya.

	Covid-19 Knowledge	Preventive Knowledge	*Covid-19 Self-action*
	β (SE)	β (SE)	β (SE)
**Age**			
15–20 years (Ref)			
21–30 years	0.09 (1.09)	0.013 (0.21)	-0.029 (-0.32)
31–40 years	0.167 (1.88)	-0.068 (-0.97)	-0.072 (-0.75)
41–50 years	0.201 (2.10)*	-0.008 (-0.11)	-0.03 (-0.30)
**Level of education**			
No formal education (Ref)			
Primary/middle school	0.212 (1.35)	0.119 (0.91)	-0.062 (-0.35)
Secondary/post primary	0.313 (1.93)	0.248 (1.83)	-0.029 (-0.16)
Tertiary/post-secondary	0.667 (4.04)***	0.229 (1.67)	-0.119 (-0.64)
**Marital status**			
Never married (Ref)			
Married/Co-habiting	0.024 (0.36)	0.019 (0.35)	-0.084 (-1.13)
Divorced/Separated/Widowed	-0.083 (-0.84)	-0.049 (-0.72)	-0.118 (-1.25)
**Rural/urban residence**			
Rural (Ref)			
Urban	0.06 (1.32)	0.227 (6.26)***	-0.132 (-2.56)*
**County**			
Bungoma (Ref)			
Kericho	-0.168 (-1.79)	0.189 (2.80)**	0.105 (1.31)
Kiambu	0.355 (3.84)***	0.024 (0.35)	0.281 (2.98)**
Kilifi	-0.054 (-0.48)	-0.301 (-3.61)***	0.149 (1.24)
Kitui	0.498 (4.96)***	0.269 (4.11)***	0.434 (4.50)***
Nairobi	0.386 (4.09)***	-0.091 (-1.31)	0.297 (3.14)**
Nandi	-0.503 (-4.76)***	0.320 (5.24)***	0.127 (1.63)
Nyamira	-0.461 (-4.38)***	0.347 (5.28)***	-0.015 (-0.17)
Siaya	0.111 (1.13)	0.143 (2.28)*	0.436 (4.30)***
Kakamega	-0.267 (-2.57)*	-0.075 (-0.90)	0.773 (7.50)***
West Pokot	-0.510 (-4.04)***	-0.164 (-1.69)	0.255 (2.09)*
**Covid-19 information**			
A little (Ref)			
Some	-0.078 (-0.54)	-0.100 (-0.90)	-0.115 (-0.71)
A lot	-0.026 (-0.20)	-0.03 (-0.29)	-0.006 (-0.04)
**Keep covid-19 secret**			
No (Ref)			
Yes	-0.127 (-1.92)	-0.262 (-3.81)***	-0.026 (-0.34)
**Know or heard of call center**			
No (Ref)			
Yes, knows the number	0.386 (4.98)***	0.237 (3.07)**	-0.011 (-0.13)
Yes, but does not know the number	0.275 (3.60)***	0.106 (1.46)	-0.059 (-0.69)
**Authorities**			
No (Ref)			
Yes	0.104 (2.11)*	0.118 (3.13)**	-0.199 (-3.74)***
**Family and friends**			
No (Ref)			
Yes	0.051 (1.11)	0.004 (0.10)	-0.006 (-0.11)
**Traditional media**			
No (Ref)			
Yes	0.305 (2.13)*	-0.083 (-0.73)	-0.250 (-1.30)
**Social media**			
No (Ref)			
Yes	0.115 (2.47)*	0.136 (3.58)***	-0.205 (-3.83)***
**Trust in family and friends**			
No (Ref)			
Yes	-0.056 (-1.04)	-0.105 (-2.72)**	0.061 (1.05)
**Trust in authorities**			
No (Ref)			
Yes	0.181 (3.03)**	0.268 (5.93)***	0.288 (4.67)***
**Trust in traditional media**			
No (Ref)			
Yes	-0.317 (-2.19)*	0.03 (0.23)	-0.165 (-0.89)
**Trust in social media**			
No (Ref)			
Yes	-0.032 (-0.59)	0.130 (3.10)**	0.045 (0.74)
Constant	5.810 (19.35)***	5.598 (24.28)***	5.984 (19.10)***
Observations	5952	5952	5952

β represents standardized coefficient

SE represents standard error

Constant ― also known as y-intercept is the mean of the dependent variable when all independent variables in the model are set to zero

* p < 0.05,

** p < 0.01,

*** p < 0.001

**Table 5:** Determinants of Covid-19 knowledge, preventive knowledge, and self-action in Nigeria.

	Covid-19 Knowledge	Preventive Knowledge	Covid-19 Self-action
	β (SE)	β (SE)	β (SE)
**Age**			
15–20 years (Ref)			
21–30 years	0.121 (0.78)	-0.104 (-0.55)	-0.229 (-1.53)
31–40 years	0.190 (1.10)	-0.391 (-1.89)	-0.192 (-1.20)
41–50 years	0.125 (0.61)	-0.404 (-1.78)	-0.316 (-1.84)
**Level of education**			
No formal education (Ref)			
Primary/middle school	0.233 (0.90)	-0.118 (-0.36)	0.193 (0.57)
Secondary/post primary	-0.069 (-0.29)	-0.182 (-0.70)	0.209 (0.65)
Tertiary/post-secondary	0.409 (1.64)	0.099 (0.35)	0.240 (0.72)
**Marital status**			
Never married (Ref)			
Married/Co-habiting	-0.244 (-1.80)	-0.055 (-0.35)	0.212 (1.84)
Divorced/Separated/Widowed	-0.533 (-2.61)**	-0.197 (-0.79)	0.041 (0.24)
**Rural/urban residence**			
Rural (Ref)			
Urban	0.177 (0.77)	0.013 (0.05)	0.621 (2.78)**
**State**			
Lagos (Ref)			
Kano	0.318 (2.15)*	-0.630 (-4.19)***	-0.835 (-7.02)***
**Covid-19 information**			
A little (Ref)			
Some	-0.105 (-0.45)	-0.115 (-0.49)	0.041 (0.21)
A lot	0.0799 (0.39)	0.448 (2.20)*	0.348 (2.07)*
**Keep covid-19 secret**			
No (Ref)			
Yes	0.077 (0.39)	-0.214 (-0.96)	-0.001 (-0.01)
**Know or heard of call center**			
No (Ref)			
Yes, knows the number	1.048 (6.41)***	0.428 (2.30)*	0.559 (3.59)***
Yes, but does not know the number	0.690 (4.61)***	0.548 (3.22)**	0.456 (3.01)**
**Authorities**			
No (Ref)			
Yes	-0.076 (-0.71)	-0.442 (-3.18)**	0.277 (2.90)**
**Family and friends**			
No (Ref)			
Yes	-0.186 (-1.85)	-0.219 (-1.89)	-0.059 (-0.75)
**Traditional media**			
No (Ref)			
Yes	0.006 (0.03)	-0.018 (-0.08)	0.003 (0.02)
**Social media**			
No (Ref)			
Yes	0.178 (1.70)	0.014 (0.12)	0.069 (0.64)
**Trust in family and friends**			
No (Ref)			
Yes	-0.175 (-1.56)	0.165 (1.21)	0.053 (0.48)
**Trust in authorities**			
No (Ref)			
Yes	0.064 (0.57)	0.056 (0.42)	-0.088 (-0.86)
**Trust in traditional media**			
No (Ref)			
Yes	0.448 (2.74)**	0.297 (1.68)	0.060 (0.51)
**Trust in social media**			
No (Ref)			
Yes	0.344 (3.33)***	-0.055 (-0.45)	0.261 (2.80)**
Constant	4.353 (12.06)***	5.670 (13.96)***	4.595 (12.17)***
Observations	1299	**1299**	1299

β represents standardized coefficient

SE represents standard error

Constant ― also known as y-intercept is the mean of the dependent variable when all independent variables in the model are set to zero

* p < 0.05,

** p < 0.01,

*** p < 0.001

Across all study countries, knowledge of COVID-19 call centre and the centre’s phone number was found to be associated with higher COVID-19 knowledge scores. Also, in Kenya (β = 0.305, SE = 2.13, p < 0.05) and the DRC (β = 0.351, SE = 2.34, p < 0.05), women that learned about COVID-19 from traditional media had higher knowledge scores than those that did not. Likewise, learning about COVID-19 from social media was associated with higher COVID-19 knowledge scores in the DRC (β = 0.111, SE = 2.29, p < 0.05) and Kenya (β = 0.115, SE = 2.47, p < 0.05). Whereas trust in authorities (β = 0.181, SE = 3.03, p < 0.01) was significantly associated with higher COVID-19 knowledge scores in Kenya, trust in traditional media (β = -0.317, SE = -2.19, p < 0.05) was associated with reduced knowledge scores. Similar patterns were observed among the sample from the DRC.

#### Factors associated with COVID-19 preventive knowledge

Tables 2–5 show the factors associated with Covid-9 preventive knowledge. Detail results on factors associated with COVID-19 preventive knowledge in can be found in S5 Table (Burkina Faso), S6 Table (the DRC), S7 Table (Kenya), and S8 Table (Nigeria). Age and education were significantly related to COVID-19 preventive knowledge in Burkina Faso. Specifically, women aged 31–40 (β = 0.492, SE = 2.58, p < 0.05) score significantly higher on COVID-19 preventive knowledge than their colleagues aged 15 to 20. Furthermore, whereas urban residents in Kenya (β = 0.227, SE = 6.26, p < 0.001) were associated with significantly higher COVID-19 preventive knowledge scores, urban residents in Burkina Faso (β = -0.411, SE = -4.32, p < 0.001) were associated with lower COVID-19 preventive knowledge scores. Having a lot of COVID-19 information (β = 0.448, SE = 2.20, p < 0.05) was associated with higher preventive knowledge scores in Nigeria. Respondents in Burkina Faso (β = 0.356, SE = 2.09, p < 0.05) that indicated that they would keep it secret if any family member contracted COVID-19 had significantly higher COVID-19 preventive knowledge compared to those that indicated otherwise. Furthermore, knowledge of a COVID-19 call centre and the phone number to call was significantly associated with COVID-19 preventive knowledge in Nigeria and Kenya. In Nigeria (β = -0.442, SE = -3.18, p < 0.01) and the DRC (β = -0.163, SE = -2.99, p < 0.01), women that learned about COVID-19 from authorities had lower COVID-19 preventive knowledge scores compared to their counterparts that did not. In contrast, women from Kenya that learned about COVID-19 from authorities (β = 0.118, SE = 3.13, p < 0.01) had significantly higher COVID-19 preventive knowledge scores than their counterparts that did not. Additionally, whereas learning about COVID-19 from social media was associated with higher preventive knowledge scores in Kenya (β = 0.136, SE = 3.58, p < 0.001), it was associated with lower preventive knowledge in the DRC (β = -0.206, SE = -3.72, p < 0.001). Trust in authorities (β = 0.512, SE = 2.30, p < 0.05) and social media (β = 0.460, SE = 3.63, p < 0.001) were associated with higher COVID-19 preventive knowledge scores in Burkina Faso. In Kenya, while trust in family and friends (β = -0.105, SE = -2.72, p < 0.01) was found to be associated with lower COVID-19 preventive knowledge scores, trust in authorities (β = 0.268, SE = 5.93, p < 0.001) and in social media (β = 0.130, SE = 3.10, p < 0.01) were found to be associated with higher COVID-19 preventive knowledge scores. In the DRC, only trust in authorities (β = 0.316, SE = 5.00, p < 0.01) was associated with higher COVID-19 preventive knowledge scores.

#### Factors associated with COVID-19 self-action

We present our findings on the determinants of COVID-19 self-action in Tables 2–5. For detail results on factors associated with COVID-19 self-action in Burkina Faso, the DRC, Kenya, and Nigeria, see S9–S12 Tables, respectively. Our regressions revealed that sociodemographic characteristics, location, and source of information influence COVID-19 self-action. For Nigeria, urban residence (β = 0.621, SE = 2.78, p < 0.01) having a lot of COVID -19 information (β = 0.348, SE = 2.07, p < 0.05), knowledge of COVID-19 call centre and the phone number (β = 0.559, SE = 3.59, p < 0.001), Knowledge of COVID -19 call centre but not its phone number (β = 0.456, SE = 3.01, p < 0.01), learning about COVID-19 from authorities (β = 0.277, SE = 2.90, p < 0.01), and trust in social media for COVID-19 information (β = 0.261, SE = 2.80, p < 0.01) were associated with higher self-action scores. In Kenya, trust in authorities (β = .288, SE = 4.67, p < 0.001) for COVID-19 information was associated with higher preventive action scores. In contrast, we found that urban residence (β = -0.132, SE = -2.56, p < 0.05), learning about COVID-19 from authorities (β = -0.199, SE = -3.74, p < 0.001) and social media (β = -0.205, SE = -3.83, p < 0.001) were associated with lower self-action scores. For Burkina Faso, respondents with secondary/post-secondary education (β = -0.227, SE = -2.06, p < 0.05) had significantly lower self-action scores than those without formal education. In addition, knowledge of COVID-19 call centre and the phone number (β = 0.512, SE = 5.20, p < 0.001) and Knowledge of COVID-19 call centre but not the phone number (β = 0.243, SE = 2.87, p < 0.01) increased COVID-19 self-action scores.

Furthermore, receipt of information from family and friends (β = 0.157, SE = 0.157, p < 0.05) and trust in social media (β = 0.288, SE = 3.87, p < 0.001) were significantly associated with higher self-action scores. Finally, in the DRC, knowledge of COVID-19 call centre and the phone number (β = 0.286, SE = 3.66, p < 0.001), receipt of information from authorities (β = 0.106, SE = 2.77, p < 0.01), receipt of information from social media (β = 0.137, SE = 3.53, p < 0.001), trust in authorities (β = 0.272, SE = 6.05, p < 0.001), and trust in social media (β = 0.136, SE = 3.28, p < 0.01) were associated with higher COVID-19 self-action scores. On the other hand, those that indicated their preparedness to keep a family COVID-19 infection secret (β = -0.210, SE = -2.94, p < 0.01) and those who trust in COVID-19 information from family and friends (β = -0.097, SE = -2.50, p < 0.05) had significantly lower COVID-19 self-action scores than their counterparts that do not.

## Discussion and conclusion

In this multi-country study, we examined the factors associated with COVID-19 knowledge, preventive knowledge, and self-action among women. Overall, our findings reveal high COVID-19 knowledge, preventive knowledge, and self-action among women across the four study countries. Other studies in the subregion have reported high COVID-19 knowledge levels and practices among different population groups [9, 50, 51]. However, variations exist across these countries. Specifically, the highest mean COVID-19 knowledge and self-action scores are from the Kenyan sample, while the highest mean preventive knowledge score is from the Nigerian sample. Despite the relatively high knowledge, preventive knowledge, and self-action scores among women in the subregion, our findings accentuate the need for enhanced efforts to improve knowledge, preventive knowledge, and self-action towards COVID-19 as the HBM and other psychosocial models suggests that increased knowledge and preventive knowledge will likely instigate preventive action [52, 53].

The multivariate analysis shows that increase in women’s age is associated with an increase in COVID-19 knowledge and preventive knowledge scores in Burkina Faso and Kenya. This finding contradicts previous studies that suggest a negative relationship between increase in age and COVID-19 knowledge [6, 24, 51]. For instance, Akalu and colleagues found increased age to be associated with poor COVID-19 knowledge among chronic disease patients in Addis Hospital in Northwest Ethiopia [24]. Similarly, Baig and colleagues found people in younger age groups better understood COVID-19 than those in older age groups [6]. Among others, the authors attribute these observations to old age induced decline in hearing and cognitive abilities. Younger adults’ risk-taking predisposition may be adduced to explain the low COVID-19 knowledge among them. Since its declaration, a wealth of evidence indicate that older adults are at a much higher risk to COVID-19 related morbidity and mortality [54]. This understanding has the potential to stimulate more older women to learn about the disease and how to prevent it.

While sociodemographic factors are important for health knowledge and self-action, their effect is greatly moderated by location, source of information, and trust in the source of information. We found that women’s location influences their COVID-19 knowledge, preventive knowledge, and self-action in the subregion. This finding conforms with earlier studies that highlight spatial variations in COVID-19 knowledge and preventive practices [24, 28]. In SSA countries, vast geographical disparities exist in access to electricity, access to health facilities where health education can be provided, access to internet services, and illiteracy rates, among several others. These developmental disparities, which are often attributed to environmental and political factors such as colonialism, can be adduced to explain the variations in our findings. For example, rural areas often lack electricity and internet services which are essential for accessing COVID-19 related information [24]. Additionally, many of these areas lack personal protective equipment (such as medical masks, goggles, gowns, gloves, and N95 respirators), which can dissuade women from acting. In China, rural residents were observed to lack appropriate COVID-19 protective materials and information, which affects their ability to act [33]. These findings highlight the need to pay attention to geography in controlling COVID-19 infections as knowledge and actions toward infectious diseases feed into the broader geographies of the subregion.

Consistent with the HBM, having access to a lot of COVID-19 information increases mean knowledge, preventive knowledge, and self-action scores. This finding is also affirmed by earlier studies [28, 50]. For example, Addis and colleagues in a study on the knowledge, attitude, and practice of patients with chronic disease towards COVID-19 pandemic in Dessie town hospital in Northeast Ethiopia found that people with poor COVID-19 knowledge were less likely to have good practice [28]. Information is vital for both COVID-19 risk and self-efficacy assessments [50]. It also enhances individuals’ understanding on ways of navigating barriers associated with seeking knowledge and acting. Relatedly, knowledge of COVID-19 call centres and the phone number led to high COVID-19 knowledge, preventive knowledge, and self-action scores among women. Following its declaration as a global pandemic, most countries established call centres to provide for the information needs of their citizenry in line with public health recommendations. Clearly, our findings demonstrate the importance of health information centres to health knowledge and behaviours and the need to scale up their operations as part of efforts to curb the spread of COVID-19.

The complex relationships between the source of information and COVID-19 knowledge and self-action are worth noting [6, 24]. We found in our analysis that different sources of information influence knowledge, preventive knowledge, and self-action scores across the study countries. This finding is in line with the health belief model which suggests that the source of information is crucial for health actions [17, 19]. Like many social issues, a wide range of information sources emerged following its declaration as a global pandemic, including media, family and friends, and authorities. Learning about COVID-19 from authorities appears to have the greatest effect on knowledge, preventive knowledge, and self-action among women. This finding is expected as authorities are often knowledge experts on the topic and hence tend to provide accurate and relevant information to people. As theorized, trust in the source of information is equally important in influencing health behaviours [17, 21]. This is very critical considering the prevalence of COVID-19 misinformation and disinformation. The effect of trust, however, depends on the source of the information and the country. For example, in Nigeria, trust in social media for COVID-19 information was found to be associated with higher COVID-19 knowledge scores but lower preventive knowledge scores. People are more likely to seek and act on health information when it is from a trusted source [7]. Generally, trust in authorities appears to have the greatest effect on COVID-19 knowledge, preventive knowledge, and self-action among women in this study. This is unsurprising considering their expertise and the authenticity of the information from authorities. These are often individuals trained in these fields with expert knowledge, and hence people tend to be influenced by input from them.

While this study makes an important contribution to the literature and efforts toward preventing and containing COVID-19 in sub-Saharan Africa, a number of limitations are worth noting. First, data for the study is cross-sectional and hence limits our ability to establish trends in associations over time. The cross-sectional nature of the data also makes it impossible to establish causal linkages between our study variables. Secondly, the study could not account for the role of risk perception in influencing knowledge, preventive knowledge, or preventive action. Finally, this study did not include older women who are at a higher risk of more severe outcomes from COVID-19 infection.

Overall, our findings suggest that the effects of social and demographic factors on health knowledge and behaviours of women in SSA in the context of COVID-19 are moderated by location, the source of information, and trust in the source of information. Hence, COVID-19 prevention measures should focus more on women’s place of residence and the source of information. Again, efforts should be made to enhance trust in approved COVID-19 information sources as that enhances knowledge levels and the propensity to act. Further qualitative exploration of the concerns of women relative to these determinants will enhance knowledge and efforts toward controlling the coronavirus pandemic.

## Supporting information

S1 Table Determinants of COVID-19 knowledge among women in Burkina Faso. (DOCX)

S2 Table Determinants of COVID-19 knowledge among women in the Democratic Republic of Congo. (DOCX)

S3 Table Determinants of COVID-19 knowledge among women in Kenya. (DOCX)

S4 Table Determinants of COVID-19 knowledge among women in Nigeria. (DOCX)

S5 Table Determinants of COVID-19 preventive knowledge among women in Burkina Faso. (DOCX)

S6 Table Determinants of COVID-19 preventive knowledge among women in the Democratic Republic of Congo. (DOCX)

S7 Table Determinants of COVID-19 preventive knowledge among women in Kenya. (DOCX)

S8 Table Determinants of COVID-19 preventive knowledge among women in Nigeria. (DOCX)

S9 Table Determinants of COVID-19 self-action among women in Burkina Faso. (DOCX)

S10 Table Determinants of COVID-19 self-action among women in the Democratic Republic of Congo. (DOCX)

S11 Table Determinants of COVID-19 self-action among women in Kenya. (DOCX)

S12 Table Determinants of COVID-19 self-action among women in Nigeria. (DOCX)
