# Supplementary material for: Determinants of COVID-19 knowledge and self-action among African women: Evidence from Burkina Faso, the Democratic Republic of Congo, Kenya, and Nigeria
Source: PLOS Glob Public Health. 2023 May 3;3(5):e0001688. doi: 10.1371/journal.pgph.0001688 (PMC10156008; doi:10.1371/journal.pgph.0001688)
Supplement: S1 Table — (DOCX) [file pgph.0001688.s001.docx]

**S1 Table: Determinants of COVID-19 knowledge among women in Burkina Faso**

|  | **Model 1** | **Model 2** | **Model 3** | **Model 4** |
| --- | --- | --- | --- | --- |
| **Variables** | β (SE) | β (SE) | β (SE) | β (SE) |
| **Age** |  |  |  |  |
| 15-20 years (Ref) |  |  |  |  |
| 21-30 years | 0.228 (1.48) | 0.128 (0.84) | 0.161 (1.06) | 0.166 (1.14) |
| 31-40 years | 0.434 (2.57)^*^ | 0.307 (1.83) | 0.321 (1.88) | 0.332 (2.05)^*^ |
| 41-50 years | 0.163 (0.88) | 0.043 (0.23) | 0.058 (0.31) | 0.093 (0.53) |
| **Level of education** |  |  |  |  |
| No formal education (Ref) |  |  |  |  |
| Primary/middle school | 0.170 (1.26) | 0.155 (1.16) | 0.098 (0.79) | 0.058 (0.48) |
| Secondary/post primary | 0.503 (2.50)^*^ | 0.413 (2.21)^*^ | 0.265 (1.56) | 0.233 (1.41) |
| Tertiary/post-secondary | 1.610 (8.11)^***^ | 1.313 (7.30)^***^ | 1.179 (6.10)^***^ | 1.150 (6.12)^***^ |
| **Marital status** |  |  |  |  |
| Never married (Ref) |  |  |  |  |
| Married/Co-habiting | 0.132 (0.54) | 0.123 (0.54) | 0.130 (0.64) | 0.120 (0.61) |
| Divorced/Separated/Widowed | 0.175 (0.55) | 0.251 (0.94) | 0.284 (1.09) | 0.281 (1.12) |
| **Rural/urban residence** |  |  |  |  |
| Rural (Ref) |  |  |  |  |
| Urban |  | 0.275 (4.23)*** | 0.194 (2.92)** | 0.180 (2.71)** |
| **County** |  |  |  |  |
| Boucle du mouhoun (Ref) |  |  |  |  |
| Cascades |  | -0.101 (-0.47) | -0.070 (-0.31) | -0.040 (-0.18) |
| Centre |  | -0.023 (-0.13) | -0.115 (-0.64) | -0.065 (-0.36) |
| Centre-est |  | -0.594 (-1.77) | -0.686 (-2.05)^*^ | -0.623 (-1.91) |
| Centre-nord |  | 0.317 (1.53) | 0.294 (1.41) | 0.398 (1.88) |
| Centre-ouest |  | -0.432 (-1.81) | -0.440 (-1.81) | -0.393 (-1.79) |
| Centre-sud |  | -0.049 (-0.20) | -0.190 (-0.77) | -0.148 (-0.60) |
| Est |  | -0.066 (-0.37) | -0.079 (-0.44) | -0.024 (-0.13) |
| Hauts-bassins |  | 0.025 (0.09) | 0.027 (0.11) | 0.043 (0.17) |
| Nord |  | -0.121 (-0.62) | -0.139 (-0.74) | -0.124 (-0.65) |
| Plateau-central |  | 0.040 (0.18) | -0.021 (-0.10) | 0.006 (0.02) |
| Sahel |  | -0.129 (-0.30) | -0.219 (-0.49) | -0.119 (-0.27) |
| Sud-ouest |  | -1.099 (-3.80)^***^ | -1.114 (-4.30)^***^ | -1.104 (-4.27)^***^ |
| **Covid-19 information** |  |  |  |  |
| A little (Ref) |  |  |  |  |
| Some |  |  | 0.230 (0.63) | 0.204 (0.49) |
| A lot |  |  | 0.209 (0.62) | 0.174 (0.44) |
| **Keep covid-19 secret** |  |  |  |  |
| No (Ref) |  |  |  |  |
| Yes |  |  | -0.178 (-0.96) | -0.184 (-1.02) |
| **Know or heard of call center** |  |  |  |  |
| No (Ref) |  |  |  |  |
| Yes, knows the number |  |  | 0.401 (2.98)^**^ | 0.356 (2.66)^**^ |
| Yes, but does not know the number |  |  | 0.121 (1.03) | 0.092 (0.79) |
| **Authorities** |  |  |  |  |
| No (Ref) |  |  |  |  |
| Yes |  |  | 0.041 (0.43) | 0.015 (0.16) |
| **Family and friends** |  |  |  |  |
| No (Ref) |  |  |  |  |
| Yes |  |  | 0.144 (1.72) | 0.145 (1.71) |
| **Traditional media** |  |  |  |  |
| No (Ref) |  |  |  |  |
| Yes |  |  | 0.173 (1.26) | 0.157 (1.17) |
| **Social media** |  |  |  |  |
| No (Ref) |  |  |  |  |
| Yes |  |  | -0.025 (-0.15) | -0.033 (-0.22) |
| **Trust in family and friends** |  |  |  |  |
| No (Ref) |  |  |  |  |
| Yes |  |  |  | -0.126 (-1.25) |
| **Trust in authorities** |  |  |  |  |
| No (Ref) |  |  |  |  |
| Yes |  |  |  | 0.419 (1.47) |
| **Trust in traditional media** |  |  |  |  |
| No (Ref) |  |  |  |  |
| Yes |  |  |  | 0.361 (1.46) |
| **Trust in social media** |  |  |  |  |
| No (Ref) |  |  |  |  |
| Yes |  |  |  | 0.099 (0.96) |
| Constant | 5.167 (17.65)*** | 5.371 (17.94)*** | 4.879 (11.26)*** | 4.245 (7.33)*** |
| Observations | 3415 | 3415 | 3415 | 3415 |

β represents standardized coefficient

SE represents standard error

Constant ― also known as y-intercept is the mean of the dependent variable when all independent variables in the model are set to zero

* p < 0.05, ** p < 0.01, *** p < 0.001
