# Supplementary material for: Determinants of COVID-19 knowledge and self-action among African women: Evidence from Burkina Faso, the Democratic Republic of Congo, Kenya, and Nigeria
Source: PLOS Glob Public Health. 2023 May 3;3(5):e0001688. doi: 10.1371/journal.pgph.0001688 (PMC10156008; doi:10.1371/journal.pgph.0001688)
Supplement: S2 Table — (DOCX) [file pgph.0001688.s002.docx]

**S2 Table: Determinants of COVID-19 knowledge among women in the Democratic Republic of Congo**

|  | **Model 1** | **Model 3** | **Model 4** |
| --- | --- | --- | --- |
| **Variables** | β (SE) | β (SE) | β (SE) |
| **Age** |  |  |  |
| 15-20 years (Ref) |  |  |  |
| 21-30 years | 0.102 (1.17) | 0.093 (1.10) | 0.095 (1.11) |
| 31-40 years | 0.198 (2.10)^*^ | 0.178 (1.96) | 0.177 (1.92) |
| 41-50 years | 0.212 (2.12)^*^ | 0.187 (1.92) | 0.189 (1.92) |
| **Level of education** |  |  |  |
| No formal education (Ref) |  |  |  |
| Primary/middle school | 0.454 (3.06)^**^ | 0.277 (1.77) | 0.269 (1.74) |
| Secondary/post primary | 0.659 (4.36)^***^ | 0.429 (2.65)^**^ | 0.416 (2.60)^**^ |
| Tertiary/post-secondary | 1.084 (7.07)^***^ | 0.834 (5.04)^***^ | 0.791 (4.83)^***^ |
| **Marital status** |  |  |  |
| Never married (Ref) |  |  |  |
| Married/Co-habiting | 0.018 (0.25) | 0.012 (0.17) | 0.015 (0.22) |
| Divorced/Separated/Widowed | -0.016 (-0.15) | -0.029 (-0.29) | -0.038 (-0.38) |
| **Covid-19 information** |  |  |  |
| A little (Ref) |  |  |  |
| Some |  | -0.161 (-1.04) | -0.149 (-0.98) |
| A lot |  | -0.096 (-0.70) | -0.062 (-0.46) |
| **Keep covid-19 secret** |  |  |  |
| No (Ref) |  |  |  |
| Yes |  | -0.135 (-1.85) | -0.117 (-1.61) |
| **Know or heard of call center** |  |  |  |
| No (Ref) |  |  |  |
| Yes, knows the number |  | 0.364 (4.63)^***^ | 0.359 (4.54)^***^ |
| Yes, but does not know the number |  | 0.335 (4.20)^***^ | 0.322 (4.04)^***^ |
| **Authorities** |  |  |  |
| No (Ref) |  |  |  |
| Yes |  | 0.149 (2.94)** | 0.109 (2.11)* |
| **Family and friends** |  |  |  |
| No (Ref) |  |  |  |
| Yes |  | 0.055 (1.16) | 0.067 (1.40) |
| **Traditional media** |  |  |  |
| No (Ref) |  |  |  |
| Yes |  | 0.188 (1.15) | 0.351 (2.34)* |
| **Social media** |  |  |  |
| No (Ref) |  |  |  |
| Yes |  | 0.097 (1.99)* | 0.111 (2.29)* |
| **Trust in family and friends** |  |  |  |
| No (Ref) |  |  |  |
| Yes |  |  | -0.094 (-1.64) |
| **Trust in authorities** |  |  |  |
| No (Ref) |  |  |  |
| Yes |  |  | 0.191 (2.87)** |
| **Trust in traditional media** |  |  |  |
| No (Ref) |  |  |  |
| Yes |  |  | -0.548 (-3.57)*** |
| **Trust in social media** |  |  |  |
| No (Ref) |  |  |  |
| Yes |  |  | -0.050 (-0.88) |
| Constant | 5.907 (35.25)*** | 5.616 (22.17)*** | 5.917 (20.47)*** |
| Observations | 5952 | 5952 | 5952 |

β represents standardized coefficient

SE represents standard error

Constant ― also known as y-intercept is the mean of the dependent variable when all independent variables in the model are set to zero

* p < 0.05, ** p < 0.01, *** p < 0.001

SE represents standard error

Constant ― also known as y-intercept is the mean of the dependent variable when all independent variables in the model are set to zero

* p < 0.05, ** p < 0.01, *** p < 0.001
