# Supplementary material for: Determinants of COVID-19 knowledge and self-action among African women: Evidence from Burkina Faso, the Democratic Republic of Congo, Kenya, and Nigeria
Source: PLOS Glob Public Health. 2023 May 3;3(5):e0001688. doi: 10.1371/journal.pgph.0001688 (PMC10156008; doi:10.1371/journal.pgph.0001688)
Supplement: S3 Table — (DOCX) [file pgph.0001688.s003.docx]

**S3 Table: Determinants of COVID-19 knowledge among women in Kenya**

|  | **Model 1** | **Model 2** | **Model 3** | **Model 4** |
| --- | --- | --- | --- | --- |
| **Variables** | β (SE) | β (SE) | β (SE) | β (SE) |
| **Age** |  |  |  |  |
| 15-20 years (Ref) |  |  |  |  |
| 21-30 years | 0.102 (1.17) | 0.09 (1.05) | 0.086 (1.04) | 0.09 (1.09) |
| 31-40 years | 0.198 (2.10)^*^ | 0.178 (1.92) | 0.165 (1.87) | 0.167 (1.88) |
| 41-50 years | 0.212 (2.12)^*^ | 0.213 (2.16)^*^ | 0.198 (2.07)^*^ | 0.201 (2.10)^*^ |
| **Level of education** |  |  |  |  |
| No formal education (Ref) |  |  |  |  |
| Primary/middle school | 0.454 (3.06)^**^ | 0.368 (2.42)^*^ | 0.218 (1.37) | 0.212 (1.35) |
| Secondary/post primary | 0.659 (4.36)^***^ | 0.533 (3.41)^***^ | 0.321 (1.96) | 0.313 (1.93) |
| Tertiary/post-secondary | 1.084 (7.07)^***^ | 0.935 (5.94)^***^ | 0.693 (4.16)^***^ | 0.667 (4.04)^***^ |
| **Marital status** |  |  |  |  |
| Never married (Ref) |  |  |  |  |
| Married/Co-habiting | 0.018 (0.25) | 0.032 (0.46) | 0.023 (0.35) | 0.024 (0.36) |
| Divorced/Separated/Widowed | -0.016 (-0.15) | -0.059 (-0.59) | -0.078 (-0.79) | -0.083 (-0.84) |
| **Rural/urban residence** |  |  |  |  |
| Rural (Ref) |  |  |  |  |
| Urban |  | 0.064 (1.41) | 0.073 (1.60) | 0.06 (1.32) |
| **County** |  |  |  |  |
| Bungoma (Ref) |  |  |  |  |
| Kericho |  | -0.147 (-1.59) | -0.176 (-1.85) | -0.168 (-1.79) |
| Kiambu |  | 0.363 (3.91)^***^ | 0.359 (3.88)^***^ | 0.355 (3.84)^***^ |
| Kilifi |  | -0.112 (-0.96) | -0.071 (-0.63) | -0.054 (-0.48) |
| Kitui |  | 0.476 (4.77)^***^ | 0.490 (4.94)^***^ | 0.498 (4.96)^***^ |
| Nairobi |  | 0.450 (4.79)^***^ | 0.419 (4.46)^***^ | 0.386 (4.09)^***^ |
| Nandi |  | -0.502 (-4.60)^***^ | -0.512 (-4.75)^***^ | -0.503 (-4.76)^***^ |
| Nyamira |  | -0.454 (-4.25)^***^ | -0.465 (-4.40)^***^ | -0.461 (-4.38)^***^ |
| Siaya |  | 0.150 (1.50) | 0.119 (1.21) | 0.111 (1.13) |
| Kakamega |  | -0.210 (-1.99)^*^ | -0.259 (-2.49)^*^ | -0.267 (-2.57)^*^ |
| West Pokot |  | -0.568 (-4.33)^***^ | -0.520 (-4.07)^***^ | -0.510 (-4.04)^***^ |
| **Covid-19 information** |  |  |  |  |
| A little (Ref) |  |  |  |  |
| Some |  |  | -0.092 (-0.62) | -0.078 (-0.54) |
| A lot |  |  | -0.05 (-0.37) | -0.026 (-0.20) |
| **Keep covid-19 secret** |  |  |  |  |
| No (Ref) |  |  |  |  |
| Yes |  |  | -0.140 (-2.12)^*^ | -0.127 (-1.92) |
| **Know or heard of call center** |  |  |  |  |
| No (Ref) |  |  |  |  |
| Yes, knows the number |  |  | 0.387 (5.01)^***^ | 0.386 (4.98)^***^ |
| Yes, but does not know the number |  |  | 0.282 (3.69)^***^ | 0.275 (3.60)^***^ |
| **Authorities** |  |  |  |  |
| No (Ref) |  |  |  |  |
| Yes |  |  | 0.141 (2.93)^**^ | 0.104 (2.11)^*^ |
| **Family and friends** |  |  |  |  |
| No (Ref) |  |  |  |  |
| Yes |  |  | 0.043 (0.98) | 0.051 (1.11) |
| **Traditional media** |  |  |  |  |
| No (Ref) |  |  |  |  |
| Yes |  |  | 0.209 (1.36) | 0.305 (2.13)^*^ |
| **Social media** |  |  |  |  |
| No (Ref) |  |  |  |  |
| Yes |  |  | 0.106 (2.29)^*^ | 0.115 (2.47)^*^ |
| **Trust in family and friends** |  |  |  |  |
| No (Ref) |  |  |  |  |
| Yes |  |  |  | -0.056 (-1.04) |
| **Trust in authorities** |  |  |  |  |
| No (Ref) |  |  |  |  |
| Yes |  |  |  | 0.181 (3.03)^**^ |
| **Trust in traditional media** |  |  |  |  |
| No (Ref) |  |  |  |  |
| Yes |  |  |  | -0.317 (-2.19)^*^ |
| **Trust in social media** |  |  |  |  |
| No (Ref) |  |  |  |  |
| Yes |  |  |  | -0.032 (-0.59) |
| Constant | 5.907 (35.25)*** | 6.041 (32.20)*** | 5.683 (21.32)*** | 5.810 (19.35)*** |
| Observations | 5952 | 5952 | 5952 | 5952 |

β represents standardized coefficient

SE represents standard error

Constant ― also known as y-intercept is the mean of the dependent variable when all independent variables in the model are set to zero

* p < 0.05, ** p < 0.01, *** p < 0.001
