# Supplementary material for: Determinants of COVID-19 knowledge and self-action among African women: Evidence from Burkina Faso, the Democratic Republic of Congo, Kenya, and Nigeria
Source: PLOS Glob Public Health. 2023 May 3;3(5):e0001688. doi: 10.1371/journal.pgph.0001688 (PMC10156008; doi:10.1371/journal.pgph.0001688)
Supplement: S4 Table — (DOCX) [file pgph.0001688.s004.docx]

**S4 Table: Determinants of COVID-19 knowledge among women in Nigeria**

|  | **Model 1** | **Model 2** | **Model 3** | **Model 4** |
| --- | --- | --- | --- | --- |
| **Variables** | β (SE) | β (SE) | β (SE) | β (SE) |
| **Age** |  |  |  |  |
| 15-20 years (Ref) |  |  |  |  |
| 21-30 years | 0.232 (1.21) | 0.187 (1.05) | 0.105 (0.66) | 0.121 (0.78) |
| 31-40 years | 0.259 (1.16) | 0.221 (1.10) | 0.130 (0.72) | 0.190 (1.10) |
| 41-50 years | 0.226 (0.92) | 0.157 (0.69) | 0.057 (0.27) | 0.125 (0.61) |
| **Level of education** |  |  |  |  |
| No formal education (Ref) |  |  |  |  |
| Primary/middle school | 0.339 (1.16) | 0.259 (0.91) | 0.213 (0.82) | 0.233 (0.90) |
| Secondary/post primary | 0.197 (0.79) | 0.100 (0.38) | -0.121 (-0.50) | -0.069 (-0.29) |
| Tertiary/post-secondary | 0.892 (3.55)^***^ | 0.799 (2.97)^**^ | 0.346 (1.35) | 0.409 (1.64) |
| **Marital status** |  |  |  |  |
| Never married (Ref) |  |  |  |  |
| Married/Co-habiting | -0.326 (-1.93) | -0.304 (-1.99)^*^ | -0.222 (-1.61) | -0.244 (-1.80) |
| Divorced/Separated/Widowed | -0.553 (-2.41)^*^ | -0.521 (-2.28)^*^ | -0.511 (-2.46)^*^ | -0.533 (-2.61)^**^ |
| **Rural/urban residence** |  |  |  |  |
| Rural (Ref) |  |  |  |  |
| Urban |  | 0.650 (2.73)^**^ | 0.260 (1.11) | 0.177 (0.77) |
| **State** |  |  |  |  |
| Lagos (Ref) |  |  |  |  |
| Kano |  | 0.411 (2.78)^**^ | 0.341 (2.26)^*^ | 0.318 (2.15)^*^ |
| **Covid-19 information** |  |  |  |  |
| A little (Ref) |  |  |  |  |
| Some |  |  | -0.142 (-0.61) | -0.105 (-0.45) |
| A lot |  |  | 0.0631 (0.31) | 0.0799 (0.39) |
| **Keep covid-19 secret** |  |  |  |  |
| No (Ref) |  |  |  |  |
| Yes |  |  | 0.071 (0.35) | 0.077 (0.39) |
| **Know or heard of call center** |  |  |  |  |
| No (Ref) |  |  |  |  |
| Yes, knows the number |  |  | 1.152 (7.02)^***^ | 1.048 (6.41)^***^ |
| Yes, but does not know the number |  |  | 0.733 (4.87)^***^ | 0.690 (4.61)^***^ |
| **Authorities** |  |  |  |  |
| No (Ref) |  |  |  |  |
| Yes |  |  | -0.053 (-0.49) | -0.076 (-0.71) |
| **Family and friends** |  |  |  |  |
| No (Ref) |  |  |  |  |
| Yes |  |  | -0.197 (-1.95) | -0.186 (-1.85) |
| **Traditional media** |  |  |  |  |
| No (Ref) |  |  |  |  |
| Yes |  |  | 0.117 (0.66) | 0.006 (0.03) |
| **Social media** |  |  |  |  |
| No (Ref) |  |  |  |  |
| Yes |  |  | 0.295 (2.87)^**^ | 0.178 (1.70) |
| **Trust in family and friends** |  |  |  |  |
| No (Ref) |  |  |  |  |
| Yes |  |  |  | -0.175 (-1.56) |
| **Trust in authorities** |  |  |  |  |
| No (Ref) |  |  |  |  |
| Yes |  |  |  | 0.064 (0.57) |
| **Trust in traditional media** |  |  |  |  |
| No (Ref) |  |  |  |  |
| Yes |  |  |  | 0.448 (2.74)^**^ |
| **Trust in social media** |  |  |  |  |
| No (Ref) |  |  |  |  |
| Yes |  |  |  | 0.344 (3.33)^***^ |
| Constant | 5.458 (20.76)^***^ | 4.910 (16.20)^***^ | 4.666 (13.31)^***^ | 4.353 (12.06)^***^ |
| Observations | 1299 | 1299 | 1299 | 1299 |

β represents the standardized coefficient

SE represents standard error

Constant ― also known as y-intercept is the mean of the dependent variable when all independent variables in the model are set to zero

* p < 0.05, ** p < 0.01, *** p < 0.001
