# Supplementary material for: Determinants of COVID-19 knowledge and self-action among African women: Evidence from Burkina Faso, the Democratic Republic of Congo, Kenya, and Nigeria
Source: PLOS Glob Public Health. 2023 May 3;3(5):e0001688. doi: 10.1371/journal.pgph.0001688 (PMC10156008; doi:10.1371/journal.pgph.0001688)
Supplement: S5 Table — (DOCX) [file pgph.0001688.s005.docx]

**S5 Table: Determinants of COVID-19 preventive knowledge among women in Burkina Faso**

|  | **Model 1** | **Model 2** | **Model 3** | **Model 4** |
| --- | --- | --- | --- | --- |
| **Variables** | β (SE) | β (SE) | β (SE) | β (SE) |
| **Age** |  |  |  |  |
| 15-20 years (Ref) |  |  |  |  |
| 21-30 years | -0.032 (-0.15) | 0.199 (1.05) | 0.227 (1.23) | 0.252 (1.44) |
| 31-40 years | 0.157 (0.68) | 0.373 (1.81) | 0.429 (2.11)^*^ | 0.492 (2.58)^*^ |
| 41-50 years | -0.102 (-0.39) | 0.156 (0.65) | 0.223 (0.93) | 0.337 (1.48) |
| **Level of education** |  |  |  |  |
| No formal education (Ref) |  |  |  |  |
| Primary/middle school | -0.255 (-1.42) | -0.081 (-0.49) | -0.059 (-0.37) | -0.195 (-1.22) |
| Secondary/post primary | -0.449 (-2.63)^**^ | -0.270 (-1.83) | -0.253 (-1.51) | -0.352 (-2.02)^*^ |
| Tertiary/post-secondary | -1.164 (-7.05)^***^ | -0.875 (-5.99)^***^ | -0.804 (-4.43)^***^ | -0.823 (-4.41)^***^ |
| **Marital status** |  |  |  |  |
| Never married (Ref) |  |  |  |  |
| Married/Co-habiting | -0.313 (-1.32) | -0.282 (-1.41) | -0.260 (-1.37) | -0.298 (-1.52) |
| Divorced/Separated/Widowed | 0.119 (0.35) | 0.064 (0.23) | 0.100 (0.37) | 0.008 (0.03) |
| **Rural/urban residence** |  |  |  |  |
| Rural (Ref) |  |  |  |  |
| Urban |  | -0.387 (-4.30)*** | -0.421 (-4.43)*** | -0.411 (-4.32)*** |
| **County** |  |  |  |  |
| Boucle du mouhoun (Ref) |  |  |  |  |
| Cascades |  | -0.304 (-0.93) | -0.235 (-0.70) | -0.205 (-0.62) |
| Centre |  | -0.430 (-1.63) | -0.444 (-1.70) | -0.329 (-1.26) |
| Centre-est |  | -1.292 (-4.44)^***^ | -1.264 (-4.22)^***^ | -1.102 (-3.71)^***^ |
| Centre-nord |  | -0.202 (-0.60) | -0.189 (-0.56) | 0.110 (0.33) |
| Centre-ouest |  | -0.921 (-2.68)^**^ | -0.987 (-2.87)^**^ | -0.934 (-2.78)^**^ |
| Centre-sud |  | -0.888 (-2.35)^*^ | -0.885 (-2.31)^*^ | -0.794 (-2.13)^*^ |
| Est |  | -0.608 (-2.12)^*^ | -0.559 (-1.95) | -0.446 (-1.55) |
| Hauts-bassins |  | -0.286 (-0.67) | -0.187 (-0.46) | -0.093 (-0.24) |
| Nord |  | -0.527 (-1.73) | -0.485 (-1.60) | -0.413 (-1.36) |
| Plateau-central |  | -0.022 (-0.07) | -0.040 (-0.12) | 0.038 (0.12) |
| Sahel |  | -0.023 (-0.05) | -0.057 (-0.13) | 0.343 (0.85) |
| Sud-ouest |  | 0.921 (2.08)^*^ | 0.945 (2.16)^*^ | 0.923 (2.09)^*^ |
| **Covid-19 information** |  |  |  |  |
| A little (Ref) |  |  |  |  |
| Some |  |  | -0.175 (-0.36) | -0.145 (-0.30) |
| A lot |  |  | -0.384 (-0.85) | -0.385 (-0.87) |
| **Keep covid-19 secret** |  |  |  |  |
| No (Ref) |  |  |  |  |
| Yes |  |  | 0.353 (2.05)* | 0.356 (2.09)* |
| **Know or heard of call center** |  |  |  |  |
| No (Ref) |  |  |  |  |
| Yes, knows the number |  |  | 0.269 (1.47) | 0.165 (0.90) |
| Yes, but does not know the number |  |  | 0.271 (1.86) | 0.204 (1.43) |
| **Authorities** |  |  |  |  |
| No (Ref) |  |  |  |  |
| Yes |  |  | -0.061 (-0.51) | -0.084 (-0.71) |
| **Family and friends** |  |  |  |  |
| No (Ref) |  |  |  |  |
| Yes |  |  | 0.118 (0.99) | 0.118 (1.00) |
| **Traditional media** |  |  |  |  |
| No (Ref) |  |  |  |  |
| Yes |  |  | -0.012 (-0.07) | 0.034 (0.19) |
| **Social media** |  |  |  |  |
| No (Ref) |  |  |  |  |
| Yes |  |  | -0.140 (-1.02) | -0.193 (-1.38) |
| **Trust in family and friends** |  |  |  |  |
| No (Ref) |  |  |  |  |
| Yes |  |  |  | 0.124 (0.80) |
| **Trust in authorities** |  |  |  |  |
| No (Ref) |  |  |  |  |
| Yes |  |  |  | 0.512 (2.30)* |
| **Trust in traditional media** |  |  |  |  |
| No (Ref) |  |  |  |  |
| Yes |  |  |  | -0.161 (-0.60) |
| **Trust in social media** |  |  |  |  |
| No (Ref) |  |  |  |  |
| Yes |  |  |  | 0.460 (3.63)*** |
| Constant | 6.393 (27.78)*** | 6.688 (21.69)*** | 6.755 (12.20)*** | 6.036 (9.71)  *** |
| Observations | 3415 | 3415 | 3415 | 3415 |

β represents standardized coefficient

SE represents standard error

Constant ― also known as y-intercept is the mean of the dependent variable when all independent variables in the model are set to zero

* p < 0.05, ** p < 0.01, *** p < 0.001
