# Supplementary material for: Determinants of COVID-19 knowledge and self-action among African women: Evidence from Burkina Faso, the Democratic Republic of Congo, Kenya, and Nigeria
Source: PLOS Glob Public Health. 2023 May 3;3(5):e0001688. doi: 10.1371/journal.pgph.0001688 (PMC10156008; doi:10.1371/journal.pgph.0001688)
Supplement: S6 Table — (DOCX) [file pgph.0001688.s006.docx]

**S6 Table: Determinants of COVID-19 preventive knowledge among women in the Democratic Republic of Congo**

|  | **Model 1** | **Model 3** | **Model 4** |
| --- | --- | --- | --- |
| **Variables** | β (SE) | β (SE) | β (SE) |
| **Age** |  |  |  |
| 15-20 years (Ref) |  |  |  |
| 21-30 years | -0.251 (-1.39) | -0.064 (-0.68) | -0.066 (-0.71) |
| 31-40 years | -0.532 (-2.28)^*^ | -0.115 (-1.14) | -0.118 (-1.19) |
| 41-50 years | -0.148 (-0.56) | -0.064 (-0.60) | -0.064 (-0.61) |
| **Level of education** |  |  |  |
| No formal education (Ref) |  |  |  |
| Primary/middle school | 0.958 (2.53)^*^ | 0.028 (0.16) | -0.026 (-0.15) |
| Secondary/post primary | 0.667 (4.39)^***^ | 0.004 (0.02) | -0.065 (-0.36) |
| Tertiary/post-secondary | 0.506 (3.25)^**^ | -0.128 (-0.69) | -0.204 (-1.12) |
| **Marital status** |  |  |  |
| Never married (Ref) |  |  |  |
| Married/Co-habiting | -0.098 (-0.59) | -0.089 (-1.14) | -0.099 (-1.29) |
| Divorced/Separated/Widowed | -0.360 (-1.27) | -0.168 (-1.70) | -0.189 (-1.93) |
| **Covid-19 information** |  |  |  |
| A little (Ref) |  |  |  |
| Some |  | -0.178 (-1.08) | -0.152 (-0.92) |
| A lot |  | -0.048 (-0.32) | -0.018 (-0.12) |
| **Keep covid-19 secret** |  |  |  |
| No (Ref) |  |  |  |
| Yes |  | -0.076 (-0.87) | -0.065 (-0.79) |
| **Know or heard of call center** |  |  |  |
| No (Ref) |  |  |  |
| Yes, knows the number |  | 0.022 (0.22) | 0.013 (0.14) |
| Yes, but does not know the number |  | -0.055 (-0.57) | -0.064 (-0.69) |
| **Authorities** |  |  |  |
| No (Ref) |  |  |  |
| Yes |  | -0.104 (-1.94) | -0.163 (-2.99)** |
| **Family and friends** |  |  |  |
| No (Ref) |  |  |  |
| Yes |  | 0.023 (0.43) | 0.010 (0.17) |
| **Traditional media** |  |  |  |
| No (Ref) |  |  |  |
| Yes |  | -0.287 (-1.34) | -0.258 (-1.19) |
| **Social media** |  |  |  |
| No (Ref) |  |  |  |
| Yes |  | -0.201 (-3.78)*** | -0.206 (-3.72)*** |
| **Trust in family and friends** |  |  |  |
| No (Ref) |  |  |  |
| Yes |  |  | 0.050 (0.82) |
| **Trust in authorities** |  |  |  |
| No (Ref) |  |  |  |
| Yes |  |  | 0.316 (5.00)*** |
| **Trust in traditional media** |  |  |  |
| No (Ref) |  |  |  |
| Yes |  |  | -0.141 (-0.74) |
| **Trust in social media** |  |  |  |
| No (Ref) |  |  |  |
| Yes |  |  | 0.040 (0.63) |
| **Constant** | 5.383 (26.75)*** | 6.367 (21.46)*** | 6.272 (19.91)*** |
| **Observations** | 5952 | 5952 | 5952 |

β represents standardized coefficient

SE represents standard error

Constant ― also known as y-intercept is the mean of the dependent variable when all independent variables in the model are set to zero

* p < 0.05, ** p < 0.01, *** p < 0.001
