# Supplementary material for: Determinants of COVID-19 knowledge and self-action among African women: Evidence from Burkina Faso, the Democratic Republic of Congo, Kenya, and Nigeria
Source: PLOS Glob Public Health. 2023 May 3;3(5):e0001688. doi: 10.1371/journal.pgph.0001688 (PMC10156008; doi:10.1371/journal.pgph.0001688)
Supplement: S7 Table — (DOCX) [file pgph.0001688.s007.docx]

**S7 Table: Determinants of COVID-19 preventive knowledge among women in Kenya**

|  | **Model 1** | **Model 2** | **Model 3** | **Model 4** |
| --- | --- | --- | --- | --- |
| **Variables** | β (SE) | β (SE) | β (SE) | β (SE) |
| **Age** |  |  |  |  |
| 15-20 years (Ref) |  |  |  |  |
| 21-30 years | 0.021 (0.30) | 0.000 (0.00) | 0.01 (0.15) | 0.013 (0.21) |
| 31-40 years | -0.040 (-0.52) | -0.081 (-1.07) | -0.076 (-1.08) | -0.068 (-0.97) |
| 41-50 years | 0.0190 (0.24) | -0.012 (-0.15) | -0.013 (-0.18) | -0.008 (-0.11) |
| **Level of education** |  |  |  |  |
| No formal education (Ref) |  |  |  |  |
| Primary/middle school | 0.517 (3.94)^***^ | 0.252 (1.88) | 0.155 (1.14) | 0.119 (0.91) |
| Secondary/post primary | 0.765 (5.68)^***^ | 0.457 (3.31)^***^ | 0.296 (2.10)^*^ | 0.248 (1.83) |
| Tertiary/post-secondary | 0.805 (5.98)^***^ | 0.486 (3.54)^***^ | 0.283 (2.00)^*^ | 0.229 (1.67) |
| **Marital status** |  |  |  |  |
| Never married (Ref) |  |  |  |  |
| Married/Co-habiting | 0.026 (0.44) | 0.036 (0.63) | 0.03 (0.54) | 0.019 (0.35) |
| Divorced/Separated/Widowed | -0.039 (-0.52) | -0.017 (-0.23) | -0.036 (-0.52) | -0.049 (-0.72) |
| **Rural/urban residence** |  |  |  |  |
| Rural (Ref) |  |  |  |  |
| Urban |  | 0.229 (6.42)^***^ | 0.242 (6.75)^***^ | 0.227 (6.26)^***^ |
| **County** |  |  |  |  |
| Bungoma (Ref) |  |  |  |  |
| Kericho |  | 0.188 (2.59)^**^ | 0.179 (2.64)^**^ | 0.189 (2.80)^**^ |
| Kiambu |  | -0.013 (-0.19) | 0.003 (0.04) | 0.024 (0.35) |
| Kilifi |  | -0.375 (-4.17)^***^ | -0.348 (-3.99)^***^ | -0.301 (-3.61)^***^ |
| Kitui |  | 0.193 (2.71)^**^ | 0.223 (3.27)^**^ | 0.269 (4.11)^***^ |
| Nairobi |  | -0.099 (-1.39) | -0.104 (-1.47) | -0.091 (-1.31) |
| Nandi |  | 0.275 (4.26)^***^ | 0.292 (4.70)^***^ | 0.320 (5.24)^***^ |
| Nyamira |  | 0.339 (4.84)^***^ | 0.346 (5.06)^***^ | 0.347 (5.28)^***^ |
| Siaya |  | 0.175 (2.64)^**^ | 0.157 (2.41)^*^ | 0.143 (2.28)^*^ |
| Kakamega |  | -0.025 (-0.29) | -0.069 (-0.83) | -0.075 (-0.90) |
| West Pokot |  | -0.274 (-2.68)^**^ | -0.208 (-2.08)^*^ | -0.164 (-1.69) |
| **Covid-19 information** |  |  |  |  |
| A little (Ref) |  |  |  |  |
| Some |  |  | -0.118 (-1.05) | -0.100 (-0.90) |
| A lot |  |  | -0.045 (-0.44) | -0.03 (-0.29) |
| **Keep covid-19 secret** |  |  |  |  |
| No (Ref) |  |  |  |  |
| Yes |  |  | -0.275 (-4.14)^***^ | -0.262 (-3.81)^***^ |
| **Know or heard of call center** |  |  |  |  |
| No (Ref) |  |  |  |  |
| Yes, knows the number |  |  | 0.246( 3.17)^**^ | 0.237 (3.07)^**^ |
| Yes, but does not know the number |  |  | 0.115 (1.57) | 0.106 (1.46) |
| **Authorities** |  |  |  |  |
| No (Ref) |  |  |  |  |
| Yes |  |  | 0.164 (4.34)^***^ | 0.118 (3.13)^**^ |
| **Family and friends** |  |  |  |  |
| No (Ref) |  |  |  |  |
| Yes |  |  | -0.003 (-0.08) | 0.004 (0.10) |
| **Traditional media** |  |  |  |  |
| No (Ref) |  |  |  |  |
| Yes |  |  | -0.065 (-0.55) | -0.083 (-0.73) |
| **Social media** |  |  |  |  |
| No (Ref) |  |  |  |  |
| Yes |  |  | 0.158 (4.22)^***^ | 0.136 (3.58)^***^ |
| **Trust in family and friends** |  |  |  |  |
| No (Ref) |  |  |  |  |
| Yes |  |  |  | -0.105 (-2.72)^**^ |
| **Trust in authorities** |  |  |  |  |
| No (Ref) |  |  |  |  |
| Yes |  |  |  | 0.268 (5.93)^***^ |
| **Trust in traditional media** |  |  |  |  |
| No (Ref) |  |  |  |  |
| Yes |  |  |  | 0.03 (0.23) |
| **Trust in social media** |  |  |  |  |
| No (Ref) |  |  |  |  |
| Yes |  |  |  | 0.130 (3.10)^**^ |
| **Constant** | 5.629 (37.51)*** | 5.821 (36.70)*** | 5.786 (27.70)*** | 5.598 (24.28)*** |
| **Observations** | 5952 | 5952 | 5952 | 5952 |

β represents standardized coefficient

SE represents standard error

Constant ― also known as y-intercept is the mean of the dependent variable when all independent variables in the model are set to zero

* p < 0.05, ** p < 0.01, *** p < 0.001
