# Supplementary material for: Determinants of COVID-19 knowledge and self-action among African women: Evidence from Burkina Faso, the Democratic Republic of Congo, Kenya, and Nigeria
Source: PLOS Glob Public Health. 2023 May 3;3(5):e0001688. doi: 10.1371/journal.pgph.0001688 (PMC10156008; doi:10.1371/journal.pgph.0001688)
Supplement: S8 Table — (DOCX) [file pgph.0001688.s008.docx]

**S8 Table: Determinants of COVID-19 preventive knowledge among women in Nigeria**

|  | **Model 1** | **Model 2** | **Model 3** | **Model 4** |
| --- | --- | --- | --- | --- |
| **Variables** | β (SE) | β (SE) | β (SE) | β (SE) |
| **Age** |  |  |  |  |
| 15-20 years (Ref) |  |  |  |  |
| 21-30 years | 0.041 (0.21) | -0.115 (-0.60) | -0.122 (-0.64) | -0.104 (-0.55) |
| 31-40 years | -0.123 (-0.56) | -0.409 (-1.98)^*^ | -0.407 (-1.98)^*^ | -0.391 (-1.89) |
| 41-50 years | -0.067 (-0.28) | -0.388 (-1.71) | -0.418 (-1.85) | -0.404 (-1.78) |
| **Level of education** |  |  |  |  |
| No formal education (Ref) |  |  |  |  |
| Primary/middle school | 0.263 (0.85) | 0.027 (0.08) | -0.075 (-0.22) | -0.118 (-0.36) |
| Secondary/post primary | 0.444 (1.88) | -0.002 (-0.01) | -0.160 (-0.61) | -0.182 (-0.70) |
| Tertiary/post-secondary | 0.867 (3.62)^***^ | 0.347 (1.26) | 0.093 (0.33) | 0.099 (0.35) |
| **Marital status** |  |  |  |  |
| Never married (Ref) |  |  |  |  |
| Married/Co-habiting | -0.187 (-1.11) | -0.045 (-0.29) | -0.071 (-0.46) | -0.055 (-0.35) |
| Divorced/Separated/Widowed | -0.232 (-0.87) | -0.198 (-0.73) | -0.229 (-0.92) | -0.197 (-0.79) |
| **Rural/urban residence** |  |  |  |  |
| Rural (Ref) |  |  |  |  |
| Urban |  | 0.110 (0.47) | -0.039 (-0.17) | 0.013 (0.05) |
| **State** |  |  |  |  |
| Lagos (Ref) |  |  |  |  |
| Kano |  | -0.603 (-4.19)^***^ | -0.609 (-4.05)^***^ | -0.630 (-4.19)^***^ |
| **Covid-19 information** |  |  |  |  |
| A little (Ref) |  |  |  |  |
| Some |  |  | -0.140 (-0.59) | -0.115 (-0.49) |
| A lot |  |  | 0.428 (2.08)^*^ | 0.448 (2.20)^*^ |
| **Keep covid-19 secret** |  |  |  |  |
| No (Ref) |  |  |  |  |
| Yes |  |  | -0.207 (-0.92) | -0.214 (-0.96) |
| **Know or heard of call center** |  |  |  |  |
| No (Ref) |  |  |  |  |
| Yes, knows the number |  |  | 0.457 (2.46)^*^ | 0.428 (2.30)^*^ |
| Yes, but does not know the number |  |  | 0.546 (3.18)^**^ | 0.548 (3.22)^**^ |
| **Authorities** |  |  |  |  |
| No (Ref) |  |  |  |  |
| Yes |  |  | -0.431 (-3.16)^**^ | -0.442 (-3.18)^**^ |
| **Family and friends** |  |  |  |  |
| No (Ref) |  |  |  |  |
| Yes |  |  | -0.190 (-1.66) | -0.219 (-1.89) |
| **Traditional media** |  |  |  |  |
| No (Ref) |  |  |  |  |
| Yes |  |  | 0.101 (0.46) | -0.018 (-0.08) |
| **Social media** |  |  |  |  |
| No (Ref) |  |  |  |  |
| Yes |  |  | 0.003 (0.03) | 0.014 (0.12) |
| **Trust in family and friends** |  |  |  |  |
| No (Ref) |  |  |  |  |
| Yes |  |  |  | 0.165 (1.21) |
| **Trust in authorities** |  |  |  |  |
| No (Ref) |  |  |  |  |
| Yes |  |  |  | 0.056 (0.42) |
| **Trust in traditional media** |  |  |  |  |
| No (Ref) |  |  |  |  |
| Yes |  |  |  | 0.297 (1.68) |
| **Trust in social media** |  |  |  |  |
| No (Ref) |  |  |  |  |
| Yes |  |  |  | -0.055 (-0.45) |
| **Constant** | 5.659 (21.48)^***^ | 6.233 (19.58)^***^ | 5.931 (15.16)^***^ | 5.670 (13.96)^***^ |
| Observations | 1299 | 1299 | 1299 | **1299** |

β represents standardized coefficient

SE represents standard error

Constant ― also known as y-intercept is the mean of the dependent variable when all independent variables in the model are set to zero

* p < 0.05, ** p < 0.01, *** p < 0.001
