# Supplementary material for: Determinants of COVID-19 knowledge and self-action among African women: Evidence from Burkina Faso, the Democratic Republic of Congo, Kenya, and Nigeria
Source: PLOS Glob Public Health. 2023 May 3;3(5):e0001688. doi: 10.1371/journal.pgph.0001688 (PMC10156008; doi:10.1371/journal.pgph.0001688)
Supplement: S9 Table — (DOCX) [file pgph.0001688.s009.docx]

**S9 Table: Determinants of COVID-19 self-action among women in Burkina Faso**

|  | **Model 1** | **Model 2** | **Model 3** | **Model 4** |
| --- | --- | --- | --- | --- |
| **Variables** | β (SE) | β (SE) | β (SE) | β (SE) |
| **Age** |  |  |  |  |
| 15-20 years (Ref) |  |  |  |  |
| 21-30 years | -0.041 (-0.35) | -0.211 (-1.97)^*^ | -0.189 (-1.86) | -0.169 (-1.66) |
| 31-40 years | 0.031 (0.25) | -0.143 (-1.23) | -0.138 (-1.28) | -0.093 (-0.84) |
| 41-50 years | -0.125 (-0.94) | -0.300 (-2.42)^*^ | -0.289 (-2.53)^*^ | -0.213 (-1.81) |
| **Level of education** |  |  |  |  |
| No formal education (Ref) |  |  |  |  |
| Primary/middle school | 0.156 (1.42) | 0.109 (1.13) | 0.068 (0.78) | -0.011 (-0.13) |
| Secondary/post primary | 0.086 (0.56) | 0.017 (0.15) | -0.161 (-1.42) | -0.227 (-2.06)^*^ |
| Tertiary/post-secondary | 0.462 (3.25)^**^ | 0.256 (2.32)^*^ | 0.070 (0.54) | 0.043 (0.34) |
| **Marital status** |  |  |  |  |
| Never married (Ref) |  |  |  |  |
| Married/Co-habiting | -0.160 (-0.86) | -0.166 (-1.19) | -0.120 (-1.02) | -0.150 (-1.31) |
| Divorced/Separated/Widowed | -0.501 (-2.20)^*^ | -0.263 (-1.21) | -0.190 (-0.96) | -0.245 (-1.29) |
| **Rural/urban residence** |  |  |  |  |
| Rural (Ref) |  |  |  |  |
| Urban |  | 0.050 (0.82) | -0.067 (-1.07) | -0.066 (-1.07) |
| **County** |  |  |  |  |
| Boucle du mouhoun (Ref) |  |  |  |  |
| Cascades |  | -0.055 (-0.25) | -0.028 (-0.14) | -0.001 (-0.01) |
| Centre |  | 0.593 (3.14)^**^ | 0.504 (2.86)^**^ | 0.579 (3.27)^**^ |
| Centre-est |  | 0.545 (2.47)^*^ | 0.457 (2.25)^*^ | 0.555 (2.76)^**^ |
| Centre-nord |  | 0.385 (1.71) | 0.361 (1.70) | 0.524 (2.48)^*^ |
| Centre-ouest |  | 0.118 (0.51) | 0.094 (0.42) | 0.132 (0.58) |
| Centre-sud |  | 0.478 (1.84) | 0.320 (1.31) | 0.385 (1.62) |
| Est |  | 0.008 (0.04) | -0.010 (-0.05) | 0.062 (0.34) |
| Hauts-bassins |  | -0.475 (-1.61) | -0.470 (-1.73) | -0.404 (-1.54) |
| Nord |  | 0.418 (1.97)^*^ | 0.411 (2.12)^*^ | 0.458 (2.37)^*^ |
| Plateau-central |  | 0.506 (2.26)^*^ | 0.434 (2.07)^*^ | 0.479 (2.25)^*^ |
| Sahel |  | -0.196 (-0.82) | -0.291 (-1.10) | -0.075 (-0.27) |
| Sud-ouest |  | -1.577 (-3.49)^***^ | -1.555 (-3.95)^***^ | -1.556 (-4.05)^***^ |
| **Covid-19 information** |  |  |  |  |
| A little (Ref) |  |  |  |  |
| Some |  |  | 0.193 (0.52) | 0.200 (0.60) |
| A lot |  |  | 0.164 (0.46) | 0.161 (0.51) |
| **Keep covid-19 secret** |  |  |  |  |
| No (Ref) |  |  |  |  |
| Yes |  |  | 0.039 (0.33) | 0.042 (0.36) |
| **Know or heard of call center** |  |  |  |  |
| No (Ref) |  |  |  |  |
| Yes, knows the number |  |  | 0.574 (5.57)^***^ | 0.512 (5.20)^***^ |
| Yes, but does not know the number |  |  | 0.282 (3.31)^***^ | 0.243 (2.87)^**^ |
| **Authorities** |  |  |  |  |
| No (Ref) |  |  |  |  |
| Yes |  |  | 0.107 (1.50) | 0.093 (1.33) |
| **Family and friends** |  |  |  |  |
| No (Ref) |  |  |  |  |
| Yes |  |  | 0.153 (2.10)* | 0.157 (0.157)* |
| **Traditional media** |  |  |  |  |
| No (Ref) |  |  |  |  |
| Yes |  |  | 0.082 (0.71) | 0.089 (0.79) |
| **Social media** |  |  |  |  |
| No (Ref) |  |  |  |  |
| Yes |  |  | 0.084 (0.78) | 0.047 (0.45) |
| **Trust in family and friends** |  |  |  |  |
| No (Ref) |  |  |  |  |
| Yes |  |  |  | 0.024 (0.29) |
| **Trust in authorities** |  |  |  |  |
| No (Ref) |  |  |  |  |
| Yes |  |  |  | 0.187 (1.10) |
| **Trust in traditional media** |  |  |  |  |
| No (Ref) |  |  |  |  |
| Yes |  |  |  | 0.089 (0.56) |
| **Trust in social media** |  |  |  |  |
| No (Ref) |  |  |  |  |
| Yes |  |  |  | 0.288 (3.87)*** |
| Constant | 5.889 (26.71)*** | 5.774 (24.82)*** | 5.210 (12.78)*** | 4.768 (11.29)*** |
| Observations | 3415 | 3415 | 3415 | 3415 |

β represents standardized coefficient

SE represents standard error

Constant ― also known as y-intercept is the mean of the dependent variable when all independent variables in the model are set to zero

* p < 0.05, ** p < 0.01, *** p < 0.001
