# Supplementary material for: Determinants of COVID-19 knowledge and self-action among African women: Evidence from Burkina Faso, the Democratic Republic of Congo, Kenya, and Nigeria
Source: PLOS Glob Public Health. 2023 May 3;3(5):e0001688. doi: 10.1371/journal.pgph.0001688 (PMC10156008; doi:10.1371/journal.pgph.0001688)
Supplement: S10 Table — (DOCX) [file pgph.0001688.s010.docx]

**S10 Table: Determinants of COVID-19 self-action among women in the Democratic Republic of Congo**

|  | **Model 1** | **Model 3** | **Model 4** |
| --- | --- | --- | --- |
| **Variables** | β (SE) | β (SE) | β (SE) |
| **Age** |  |  |  |
| 15-20 years (Ref) |  |  |  |
| 21-30 years | 0.021 (0.30) | 0.022 (0.33) | 0.021 (0.33) |
| 31-40 years | -0.040 (-0.52) | -0.047 (-0.66) | -0.044 (-0.62) |
| 41-50 years | 0.019 (0.24) | 0.009 (0.12) | 0.010 (0.14) |
| **Level of education** |  |  |  |
| No formal education (Ref) |  |  |  |
| Primary/middle school | 0.517 (3.94)^***^ | 0.37o (2.73)^**^ | 0.313 (2.40)^*^ |
| Secondary/post primary | 0.765 (5.68)^***^ | 0.551 (3.93)^***^ | 0.479 (3.54)^***^ |
| Tertiary/post-secondary | 0.805 (5.98)^***^ | 0.552 (3.90)^***^ | 0.475 (3.47)^***^ |
| **Marital status** |  |  |  |
| Never married (Ref) |  |  |  |
| Married/Co-habiting | 0.026 (0.44) | 0.027 (0.48) | 0.017 (0.30) |
| Divorced/Separated/Widowed | -0.039 (-0.52) | -0.046 (-0.65) | -0.060 (-0.85) |
| **Covid-19 information** |  |  |  |
| A little (Ref) |  |  |  |
| Some |  | -0.121 (-1.05) | -0.102 (-0.90) |
| A lot |  | -0.069 (-0.66) | -0.052 (-0.49) |
| **Keep covid-19 secret** |  |  |  |
| No (Ref) |  |  |  |
| Yes |  | -0.228 (-3.33)*** | -0.210 (-2.94)** |
| **Know or heard of call center** |  |  |  |
| No (Ref) |  |  |  |
| Yes, knows the number |  | 0.301 (3.87)^***^ | 0.286 (3.66)^***^ |
| Yes, but does not know the number |  | 0.158 (2.09)^*^ | 0.148 (1.95) |
| **Authorities** |  |  |  |
| No (Ref) |  |  |  |
| Yes |  | 0.154 (4.01)*** | 0.106 (2.77)** |
| **Family and friends** |  |  |  |
| No (Ref) |  |  |  |
| Yes |  | -0.029 (-0.77) | -0.024 (-0.63) |
| **Traditional media** |  |  |  |
| No (Ref) |  |  |  |
| Yes |  | 0.006 (0.04) | -0.014 (-0.12) |
| **Social media** |  |  |  |
| No (Ref) |  |  |  |
| Yes |  | 0.160 (4.19)*** | 0.137 (3.53)*** |
| **Trust in family and friends** |  |  |  |
| No (Ref) |  |  |  |
| Yes |  |  | -0.097 (-2.50)* |
| **Trust in authorities** |  |  |  |
| No (Ref) |  |  |  |
| Yes |  |  | 0.272 (6.05)*** |
| **Trust in traditional media** |  |  |  |
| No (Ref) |  |  |  |
| Yes |  |  | 0.004 (0.03) |
| **Trust in social media** |  |  |  |
| No (Ref) |  |  |  |
| Yes |  |  | 0.136 (3.28)** |
| Constant | 5.629 (37.51)*** | 5.571 (27.45)*** | 5.434 (23.77)*** |
| Observations | 5952 | 5952 | 5952 |

β represents standardized coefficient

SE represents standard error

Constant ― also known as y-intercept is the mean of the dependent variable when all independent variables in the model are set to zero

* p < 0.05, ** p < 0.01, *** p < 0.001
