# Supplementary material for: Determinants of COVID-19 knowledge and self-action among African women: Evidence from Burkina Faso, the Democratic Republic of Congo, Kenya, and Nigeria
Source: PLOS Glob Public Health. 2023 May 3;3(5):e0001688. doi: 10.1371/journal.pgph.0001688 (PMC10156008; doi:10.1371/journal.pgph.0001688)
Supplement: S11 Table — (DOCX) [file pgph.0001688.s011.docx]

**S11 Table: Determinants of COVID-19 self-action among women in Kenya**

|  | **Model 1** | **Model 2** | **Model 3** | **Model 4** |
| --- | --- | --- | --- | --- |
| **Variables** | β (SE) | β (SE) | β (SE) | β (SE) |
| **Age** |  |  |  |  |
| 15-20 years (Ref) |  |  |  |  |
| 21-30 years | -0.088 (-0.92) | -0.056 (-0.61) | -0.036 (-0.39) | -0.029 (-0.32) |
| 31-40 years | -0.148 (-1.44) | -0.110 (-1.11) | -0.080 (-0.82) | -0.072 (-0.75) |
| 41-50 years | -0.092 (-0.86) | -0.066 (-0.64) | -0.037 (-0.37) | -0.03 (-0.30) |
| **Level of education** |  |  |  |  |
| No formal education (Ref) |  |  |  |  |
| Primary/middle school | -0.042 (-0.24) | -0.108 (-0.60) | -0.033 (-0.18) | -0.062 (-0.35) |
| Secondary/post primary | -0.099 (-0.56) | -0.106 (-0.57) | 0.009 (0.04) | -0.029 (-0.16) |
| Tertiary/post-secondary | -0.254 (-1.43) | -0.215 (-1.15) | -0.076 (-0.40) | -0.119 (-0.64) |
| **Marital status** |  |  |  |  |
| Never married (Ref) |  |  |  |  |
| Married/Co-habiting | -0.090 (-1.14) | -0.073 (-0.95) | -0.070 (-0.93) | -0.084 (-1.13) |
| Divorced/Separated/Widowed | -0.172 (-1.74) | -0.104 (-1.08) | -0.096 (-1.00) | -0.118 (-1.25) |
| **Rural/urban residence** |  |  |  |  |
| Rural (Ref) |  |  |  |  |
| Urban |  | -0.108 (-2.10)^*^ | -0.112 (-2.18)^*^ | -0.132 (-2.56)^*^ |
| **County** |  |  |  |  |
| Bungoma (Ref) |  |  |  |  |
| Kericho |  | 0.100 (1.24) | 0.107 (1.32) | 0.105 (1.31) |
| Kiambu |  | 0.291 (3.12)^**^ | 0.270 (2.86)^**^ | 0.281 (2.98)^**^ |
| Kilifi |  | 0.123 (1.08) | 0.094 (0.81) | 0.149 (1.24) |
| Kitui |  | 0.372 (3.75)^***^ | 0.378 (3.85)^***^ | 0.434 (4.50)^***^ |
| Nairobi |  | 0.298 (3.19)^**^ | 0.305 (3.29)^**^ | 0.297 (3.14)^**^ |
| Nandi |  | 0.121 (1.54) | 0.113 (1.42) | 0.127 (1.63) |
| Nyamira |  | -0.018 (-0.20) | -0.017 (-0.19) | -0.015 (-0.17) |
| Siaya |  | 0.425 (4.13)^***^ | 0.437 (4.31)^***^ | 0.436 (4.30)^***^ |
| Kakamega |  | 0.779 (7.29)^***^ | 0.787 (7.55)^***^ | 0.773 (7.50)^***^ |
| West Pokot |  | 0.203 (1.69) | 0.205 (1.69) | 0.255 (2.09)^*^ |
| **Covid-19 information** |  |  |  |  |
| A little (Ref) |  |  |  |  |
| Some |  |  | -0.142 (-0.87) | -0.115 (-0.71) |
| A lot |  |  | -0.038 (-0.26) | -0.006 (-0.04) |
| **Keep covid-19 secret** |  |  |  |  |
| No (Ref) |  |  |  |  |
| Yes |  |  | -0.031 (-0.40) | -0.026 (-0.34) |
| **Know or heard of call center** |  |  |  |  |
| No (Ref) |  |  |  |  |
| Yes, knows the number |  |  | -0.013 (-0.14) | -0.011 (-0.13) |
| Yes, but does not know the number |  |  | -0.053 (-0.60) | -0.059 (-0.69) |
| **Authorities** |  |  |  |  |
| No (Ref) |  |  |  |  |
| Yes |  |  | -0.146 (-2.78)^**^ | -0.199 (-3.74)^***^ |
| **Family and friends** |  |  |  |  |
| No (Ref) |  |  |  |  |
| Yes |  |  | 0.008 (0.16) | -0.006 (-0.11) |
| **Traditional media** |  |  |  |  |
| No (Ref) |  |  |  |  |
| Yes |  |  | -0.296 (-1.55) | -0.250 (-1.30) |
| **Social media** |  |  |  |  |
| No (Ref) |  |  |  |  |
| Yes |  |  | -0.199 (-3.86)^***^ | -0.205 (-3.83)^***^ |
| **Trust in family and friends** |  |  |  |  |
| No (Ref) |  |  |  |  |
| Yes |  |  |  | 0.061 (1.05) |
| **Trust in authorities** |  |  |  |  |
| No (Ref) |  |  |  |  |
| Yes |  |  |  | 0.288 (4.67)^***^ |
| **Trust in traditional media** |  |  |  |  |
| No (Ref) |  |  |  |  |
| Yes |  |  |  | -0.165 (-0.89) |
| **Trust in social media** |  |  |  |  |
| No (Ref) |  |  |  |  |
| Yes |  |  |  | 0.045 (0.74) |
| Constant | 5.995 (30.59)*** | 5.706 (27.80*** | 6.105 (20.99)*** | 5.984 (19.10)^***^ |
| Observations | 5952 | 5952 | 5952 | 5952 |

β represents standardized coefficient

SE represents standard error

Constant ― also known as y-intercept is the mean of the dependent variable when all independent variables in the model are set to zero

* p < 0.05, ** p < 0.01, *** p < 0.001
