# Supplementary material for: Determinants of COVID-19 knowledge and self-action among African women: Evidence from Burkina Faso, the Democratic Republic of Congo, Kenya, and Nigeria
Source: PLOS Glob Public Health. 2023 May 3;3(5):e0001688. doi: 10.1371/journal.pgph.0001688 (PMC10156008; doi:10.1371/journal.pgph.0001688)
Supplement: S12 Table — (DOCX) [file pgph.0001688.s012.docx]

**S12 Table: Determinants of COVID-19 self-action among women in Nigeria**

|  | **Model 1** | **Model 2** | **Model 3** | **Model 4** |
| --- | --- | --- | --- | --- |
| **Variables** | β (SE) | β (SE) | β (SE) | β (SE) |
| **Age** |  |  |  |  |
| 15-20 years (Ref) |  |  |  |  |
| 21-30 years | 0.150 (0.87) | -0.212 (-1.44) | -0.247 (-1.68) | -0.229 (-1.53) |
| 31-40 years | 0.428 (2.40)^*^ | -0.191 (-1.19) | -0.236 (-1.49) | -0.192 (-1.20) |
| 41-50 years | 0.413 (2.21)^*^ | -0.310 (-1.81) | -0.358 (-2.08)^*^ | -0.316 (-1.84) |
| **Level of education** |  |  |  |  |
| No formal education (Ref) |  |  |  |  |
| Primary/middle school | 0.800 (2.16)^*^ | 0.241 (0.69) | 0.202 (0.61) | 0.193 (0.57) |
| Secondary/post primary | 1.328 (3.88)^***^ | 0.325 (0.98) | 0.203 (0.63) | 0.209 (0.65) |
| Tertiary/post-secondary | 1.645 (4.80)^***^ | 0.496 (1.48) | 0.219 (0.66) | 0.240 (0.72) |
| **Marital status** |  |  |  |  |
| Never married (Ref) |  |  |  |  |
| Married/Co-habiting | 0.198 (1.68) | -0.073 (-0.95) | 0.216 (1.86) | 0.212 (1.84) |
| Divorced/Separated/Widowed | 0.057 (0.32) | -0.104 (-1.08) | 0.032 (0.19) | 0.041 (0.24) |
| **Rural/urban residence** |  |  |  |  |
| Rural (Ref) |  |  |  |  |
| Urban |  | 0.883 (3.91)*** | 0.640 (2.83)^**^ | 0.621 (2.78)^**^ |
| **State** |  |  |  |  |
| Lagos (Ref) |  |  |  |  |
| Kano |  | -0.805 (-6.75)^***^ | -0.858 (-7.22)^***^ | -0.835 (-7.02)^***^ |
| **Covid-19 information** |  |  |  |  |
| A little (Ref) |  |  |  |  |
| Some |  |  | 0.025 (0.13) | 0.041 (0.21) |
| A lot |  |  | 0.337 (2.01)^*^ | 0.348 (2.07)^*^ |
| **Keep covid-19 secret** |  |  |  |  |
| No (Ref) |  |  |  |  |
| Yes |  |  | -0.008 (-0.06) | -0.001 (-0.01) |
| **Know or heard of call center** |  |  |  |  |
| No (Ref) |  |  |  |  |
| Yes, knows the number |  |  | 0.607 (4.11)^***^ | 0.559 (3.59)^***^ |
| Yes, but does not know the number |  |  | 0.477 (3.21)^**^ | 0.456 (3.01)^**^ |
| **Authorities** |  |  |  |  |
| No (Ref) |  |  |  |  |
| Yes |  |  | 0.269 (3.01)^**^ | 0.277 (2.90)^**^ |
| **Family and friends** |  |  |  |  |
| No (Ref) |  |  |  |  |
| Yes |  |  | -0.053 (-0.67) | -0.059 (-0.75) |
| **Traditional media** |  |  |  |  |
| No (Ref) |  |  |  |  |
| Yes |  |  | -0.003 (-0.02) | 0.003 (0.02) |
| **Social media** |  |  |  |  |
| No (Ref) |  |  |  |  |
| Yes |  |  | 0.151 (1.33) | 0.069 (0.64) |
| **Trust in family and friends** |  |  |  |  |
| No (Ref) |  |  |  |  |
| Yes |  |  |  | 0.053 (0.48) |
| **Trust in authorities** |  |  |  |  |
| No (Ref) |  |  |  |  |
| Yes |  |  |  | -0.088 (-0.86) |
| **Trust in traditional media** |  |  |  |  |
| No (Ref) |  |  |  |  |
| Yes |  |  |  | 0.060 (0.51) |
| **Trust in social media** |  |  |  |  |
| No (Ref) |  |  |  |  |
| Yes |  |  |  | 0.261 (2.80)^**^ |
| Constant | 4.507 (12.93)*** | 5.114 (13.03)^***^ | 4.719 (12.57)^***^ | 4.595 (12.17)^***^ |
| Observations | 1299 | 1299 | 1299 | 1299 |

β represents standardized coefficient

SE represents standard error

Constant ― also known as y-intercept is the mean of the dependent variable when all independent variables in the model are set to zero

* p < 0.05, ** p < 0.01, *** p < 0.001
